# Digital Interventions Targeting Parents to Improve Early Childhood Movement, Nutrition, and Sleep Behaviors: Systematic Review

**DOI:** 10.2196/85525

**Published:** 2026-06-26

**Authors:** Johanna Sandborg, Brittany L Reese, Sarah Marshall, Kylie D Hesketh, Rachel Laws, Katherine L Downing

**Affiliations:** 1 Institute for Physical Activity and Nutrition (IPAN), Faculty of Health, Deakin University, Locked Bag 20000, Geelong, VIC, 3220, Australia, +46 733285912; 2 Sydney School of Public Health, The University of Sydney, Sydney, Australia

**Keywords:** mHealth, movement behaviours, sedentary behaviour, screen time, physical activity, breastfeeding, diet, digital, eHealth

## Abstract

**Background:**

Early childhood (0‐5 years) is key for shaping health behaviors, yet optimal behaviors are rarely achieved. Digital health promotion interventions offer scalable support for families; however, most research has focused on childhood more broadly, leaving limited evidence for the early childhood period.

**Objective:**

The primary aim of this systematic review was to examine whether autonomously delivered digital interventions targeting parents are effective at increasing physical activity, reducing sedentary behavior, improving nutrition (breastfeeding, feeding practices), and/or optimizing sleep among children aged 0‐5 years. The secondary aim was to review the reporting of co-design practices, user engagement, and process evaluation, and to assess how engagement influences intervention effectiveness.

**Methods:**

Seven databases were searched for randomized controlled trials (RCTs) evaluating autonomously delivered digital interventions targeting one or more of the following behaviors: physical activity, sedentary behavior, nutrition, or sleep among children (published to January 2026). Study quality was assessed using the Joanna Briggs Institute Critical Appraisal Checklist for RCTs. Findings were narratively synthesized by target age-group and behavior, and the direction of effect was summarized in structured tables.

**Results:**

Of the 14,352 identified records, 38 interventions (33 RCTs, 4 pilot RCTs, and 1 feasibility RCT) were included. Most studies focused on pregnancy to infancy (n=24; 0‐1 y), followed by preschoolers (n=8; 3‐5 y) and toddlers (n=6; 1‐2 y). Intervention duration ranged from 2 weeks to 1000 days, and various digital formats were used (apps n=11, SMS text messaging n=10, web- or internet-based platforms n=6, WeChat [Tencent] n=3, tablet-based program n=2, a combination of app and SMS text messaging n=1, website and emails n=1, emails and SMS text messaging n=1, automated voice calls n=1, Facebook Messenger Chatbot [Meta] n=1, and online videos n=1). Interventions spanning pregnancy to infancy reported mixed findings for breastfeeding and feeding practices. Studies targeting toddlers showed improvements in sleep, mixed findings for diet and screen time, and no differences in physical activity. Most studies targeting preschoolers reported significant improvements for feeding practices and diet, but no differences in physical activity, sedentary behavior and sleep, and mixed findings for screen time. Most studies reported co-design or engagement (n=24), but few examined the impact of engagement on intervention effectiveness (n=6), and those that did reported mixed findings. Interpretation was limited by heterogeneous designs, inconsistent outcome measures, and mixed risk-of-bias ratings across studies.

**Conclusions:**

This review advances the field by synthesizing evidence on scalable digital interventions that support parents in promoting healthy lifestyle behaviors across the first 2000 days, together with key design and implementation factors that have rarely been reported in previous reviews. Unlike prior work, it focuses exclusively on autonomously delivered digital interventions in early childhood. Findings show heterogeneous designs and mixed effectiveness, and highlight 3 priority evidence gaps: limited studies in toddlers and preschoolers, incomplete reporting of engagement, and limited understanding of how engagement influences outcomes. These findings define priorities for future research to strengthen the evidence for scalable digital interventions in early childhood.

## Introduction

### Background

Early childhood (birth through 5 years) is recognized as a critical period during which key health behaviors (diet, physical activity, sedentary behavior, and sleep) are established. However, evidence shows that these behaviors are suboptimal from early life. Despite the well-established benefits of breastfeeding, fewer than half of infants are exclusively breastfed for the first 6 months [1]. Similarly, global evidence indicates that adherence to early childhood dietary [2-4] and movement behavior guidelines is low, with large international reviews reporting that only a small proportion of children meet recommendations across physical activity, sedentary behavior, screen time, and sleep [2,3]. Of particular concern is the fact that these suboptimal behaviors can track into later childhood and adolescence [4,5], underscoring the need for interventions to promote health behaviors from a young age. Recent studies have also highlighted widening socioeconomic inequalities in children’s early environments [6,7] and less optimal diet, physical activity, sedentary behavior, screen time, and sleep among children from socioeconomically disadvantaged backgrounds [8-11], reinforcing the need for accessible intervention strategies for families who may have limited access to traditional face-to-face services.

Existing early childhood interventions have shown varied success in improving diet, physical activity, sedentary behavior, and sleep habits [12-15]. Traditionally, these interventions have relied largely on time-consuming and costly face-to-face delivery, with limited consideration of scalability or implementation at scale [16]. In contrast, digital interventions (eHealth and mobile health [mHealth]) have the advantage that they can be delivered anywhere, anytime, maximizing potential reach across diverse socioeconomic, geographical, and cultural backgrounds. Recent years have seen a rapid expansion of digital health solutions, including web-based platforms, mobile apps, and wearable technologies [17], supported by growing evidence of their potential for scalability and cost-effectiveness [18,19].

Reflecting this broader growth in digital health, there has also been a marked increase in digital interventions targeting diet and movement behaviors across all age groups, with most reporting efficacy in changing behavior [20-22]. This also includes a growing interest in the feasibility and effectiveness of these types of interventions for targeting childhood obesity and obesity-related behaviors [23-31]. However, these reviews have largely focused on childhood broadly (0‐18 years) [23,24,27-30] or have focused solely on preschoolers (3‐6 years) [25,26]. Many have also examined single behavioral domains such as physical activity or sedentary behavior [27-29], or specific population groups such as Indigenous mothers of young children [31]. In addition, most evaluate digital interventions that involve some degree of human support or multicomponent programs and do not address key design and implementation factors. As such, they offer limited insight into early childhood as a distinct developmental period or the potential of autonomously delivered interventions to support parents across multiple behaviors in the first 2000 days. Given that health behaviors are largely shaped early in life and that this life stage is characterized by unique developmental and parental influence, a review focusing solely on this period is warranted.

Moreover, co-design, engagement, and implementation factors are highly important for successful intervention delivery [32,33]; yet, these elements remain inconsistently assessed and underreported in interventions targeting parents of young children [34]. Thus, this review provides a timely and comprehensive synthesis of autonomously delivered digital interventions targeting parents across the first 2000 days (conception to age 5 years), examining multiple behavioral domains (breastfeeding, feeding practices, diet, physical activity, sedentary behavior, screen time, and sleep) and integrating evidence on co-design, process evaluation, and engagement. This broader scope allows us to identify key gaps, emerging patterns and implications for the design and implementation of scalable digital strategies in early childhood.

### Objectives

The primary aim of this systematic review was to examine whether autonomously delivered digital interventions targeting parents are effective at increasing physical activity, reducing sedentary behavior, improving nutrition (breastfeeding, feeding practices, and dietary outcomes), and/or optimizing sleep among children aged 0‐5 years. The secondary aim was to review the reporting of co-design practices, user engagement, and process evaluation, and to assess how engagement influences intervention effectiveness.

## Methods

### Eligibility Criteria

We included randomized controlled trials (RCTs) of interventions delivered to parents and caregivers of children in the first 2000 days of life (herein referred to as parents) solely via digital technology (mHealth/eHealth). Interventions needed to aim to improve one or more of the following behaviors: increase physical activity, reduce sedentary behavior, improve nutrition (breastfeeding, food intake, and feeding practices), and/or optimize sleep among children.

We only included interventions that solely used digital technologies to autonomously deliver the intervention (ie, where no personnel were needed to deliver and/or maintain the intervention). We used this definition as we were interested in solutions for intervention delivery that could be more easily and cost-effectively scaled compared to interventions requiring delivery personnel (with or without a digital component). Therefore, we excluded digital interventions that required delivery personnel for one-to-one support, for example, telephone coaching calls or face-to-face counseling sessions with a supplementary online social support group.

We considered direct outcome measures of children’s target behaviors (eg, parent-reported or accelerometer-measured physical activity) and indirect outcome measures known to influence children’s target behaviors (eg, parental feeding style or changes to food environment). There were 2 key reasons we decided to also include indirect outcome measures: (1) it can be difficult to accurately measure child behaviors, particularly for younger children (eg, breastmilk intake), and (2) national guidelines for infant feeding [35] and movement behaviors in the early years [36] include parental influences known to impact child behaviors (eg, for establishing healthy sleep habits, parents can set up a calming bedtime routine and consistent sleep and wake-up times).

Studies were also excluded if they were (1) not an RCT design, (2) solely targeting other caregivers (eg, grandparents or childcare providers), (3) among parents with older children (≥6 years old), (4) among parents with children who had clinical health conditions (eg, diabetes and premature birth), and (5) interventions delivered primarily within the antenatal period (noting that those delivered from pregnancy to infancy were included). We limited studies to primary research published in English in the peer-reviewed literature. Literature reviews and meta-analyses, theses, conference proceedings, and gray literature were not included.

### Information Sources

We searched 7 electronic databases, including Embase (Elsevier), Academic Search Complete (EBSCO), CINAHL Complete (EBSCO), Global Health, MEDLINE Complete (EBSCO), PsycINFO (EBSCO), and SPORTDiscus (EBSCO). All databases were searched individually rather than simultaneously via a multidatabase platform. We did not search study registries, conference proceedings, websites, or other online sources, nor did we undertake citation searching or contact authors or experts. No supplementary search methods beyond database searching were used, as the review was limited to peer-reviewed RCTs. Searches were conducted in December 2022 and updated in August 2024 and again in January 2026.

### Search Strategy

This systematic review was prospectively registered with PROSPERO (International Prospective Register of Systematic Reviews; ID: CRD42022372639) [37]. We made 2 amendments to the registered protocol: (1) to limit study design to RCTs due to the recent increase in publications and (2) the use of a different quality appraisal tool specific to RCTs. The PRISMA (Preferred Reporting Items for Systematic Reviews and Meta-Analysis) 2020 statement guidelines [38] and the PRISMA-S (Preferred Reporting Items for Systematic Reviews and Meta-Analyses Literature Search Extension; Checklist 1) extension for reporting search strategies were followed to ensure transparent reporting of the search process [39].

The search strategy was developed by BM, SM, and KD, in consultation with all authors. We ran preliminary searches and sought technical guidance from Deakin University librarians to refine the search strategy. The search strategy included a combination of keywords to capture concepts according to the Population, Intervention, Comparison, Outcomes, and Study design (PICOS) tool: (1) children aged ≤5 years, (2) mHealth or eHealth intervention, (3) intervention behavioral targets (ie, child physical activity, sedentary behavior, nutrition, and/or sleep), and (4) study design (see Multimedia Appendix 1). The search strategy was not adapted from previous reviews and did not undergo a formal peer-review process.

### Selection Process

All search results were exported to Covidence and duplicates were removed. Two reviewers (SM or JS and either BM, KD, or KH) independently screened all articles first by the title and abstract; a third independent reviewer resolved discrepancies (BM or KD). Two reviewers screened the full texts (BM and SM or JS and KD) using the inclusion/exclusion criteria described above. Discrepancies were resolved by a third reviewer (KD or KH). During the updated review in 2026, 2 researchers, CS and SR, assisted with screening, data extraction, and evaluation (disagreements were resolved by JS) following the same procedure.

### Data Collection Process

Two reviewers (JS and BM or CS and SR) extracted the following information using a prepiloted data collection template developed for this review. Template fields included study characteristics (eg, authors, year of publication, country, study design, study aims, and target behaviors), setting and participants (eg, setting, inclusion/exclusion criteria, recruitment, and sample size), intervention description (eg, technology used, delivery mode, content, onboarding processes, and engagement), and participant outcomes (eg, measures related to target behavioral outcomes, data collection tool and method, and results). Co-design (eg, stakeholder engagement in intervention development), intervention theory (ie, the use of a behavior change theory in intervention development), process evaluation outcomes (eg, acceptability, feasibility, and reach), and intervention engagement data (eg, app analytics, self-reported use, and impact of engagement on intervention effectiveness) were extracted using a combination of the data extraction tool at Elicit.com [40] (Ought; an online platform that automates data extraction) and manual extraction and checked for accuracy and completeness by one author (KD or CS).

### Data Items

We extracted data on all relevant behavioral outcomes (breastfeeding, feeding practices, diet, physical activity, sedentary behavior, screen time, and sleep). For each outcome, all reported time points and measurement tools were collected when available. We also extracted additional study variables, including participant characteristics, intervention features, delivery mode, co-design processes, theoretical underpinnings, process evaluation outcomes, and engagement metrics. When information was unclear or missing, assumptions were not made; instead, data were recorded as reported.

### Study Risk of Bias Assessment

The risk of bias and quality assessment for each individual study was assessed by 2 authors independently (JS and KD or CS and SR) using the Joanna Briggs Institute (JBI) Critical Appraisal Checklist for RCTs [41]. The checklist includes 13 items, where each item can be scored yes, no, unclear, or not applicable, in the categories of selection and allocation; administration of intervention/exposure; assessment, detection, and measurement of outcomes; participant retention; and statistical conclusion validity. As the interventions were autonomously delivered (eg, via digital platforms), there were no treatment deliverers involved; therefore, the item “Treatment deliverers blinded” was not applicable in this context. The initial interrater agreement between JS and KD was 81%, and 85% for CS and SR. Discrepancies in assessment between authors were discussed (JS and KD or CS and SR) until consensus was reached.

### Synthesis Methods

Consistent with Cochrane guidance [42], due to the heterogeneity in definition, measurement, and reporting of outcomes across studies, meaning they did not estimate the same underlying effect, a meta-analysis was not able to be conducted. Therefore, following PRISMA [38] and synthesis without meta-analysis recommendations [43], a structured narrative synthesis was undertaken, grouping studies by target age group (pregnancy-infancy, toddlers, and preschoolers) and by behavioral domain (breastfeeding, diet, physical activity, sedentary behavior, and sleep). We summarized the direction of effect and patterns in effectiveness using structured tables.

### Certainty Assessment

We did not conduct a formal certainty-of-evidence assessment because of the heterogeneity of the included studies and the lack of commensurable effect estimates. Instead, we considered study-level risk of bias and the consistency of findings when interpreting the results.

## Results

### Study Selection

**Figure 1.**
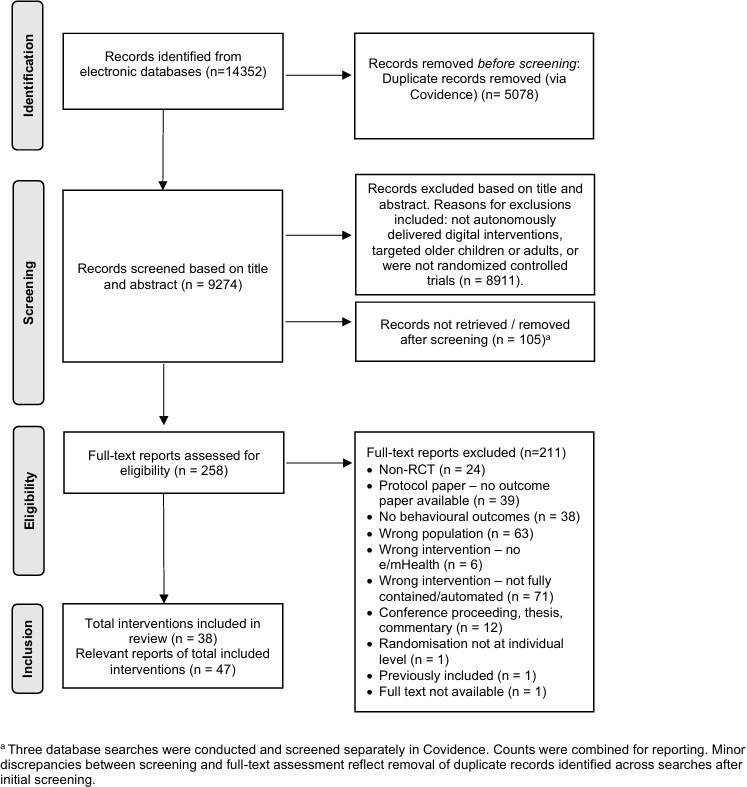
Preferred Reporting Items for Systematic Reviews and Meta-Analyses (PRISMA) flow diagram showing the study selection for randomized controlled trials of autonomously delivered digital interventions targeting early childhood health behaviors (0‐5 years). Studies involved healthy parent-child populations across multiple countries and were identified through searches conducted in December 2022, August 2024, and January 2026. mHealth: mobile health; RCT: randomized controlled trial.

The literature search yielded 14,352 unique articles. Most records excluded at the title and abstract stage were due to at least one of the following reasons: the study did not examine an autonomously delivered digital intervention (eg, clinical-delivered or face-to-face), targeted populations outside the scope of the review (eg, older children, adolescents, or adults), or did not use an RCT design (eg, observational studies, qualitative studies, pilot feasibility work without randomization, or large noninterventional epidemiological analyses). Following screening, 258 full-text reports were assessed for eligibility, of which 49 studies describing 38 interventions were included in this review. No studies were excluded at full-text review that appeared to meet the inclusion criteria; all exclusions were based on predefined criteria (eg, wrong age group, design, and intervention type). The screening process is presented in Figure 1.

### Study Characteristics

An overview of the targeted outcomes and age groups for the 38 included interventions [44-81] is presented in Table 1. Almost half of the included studies (50%, 19/38) [45,48,51,53,55,56,58,59,62,64,65,67,69,70,72,76,77,79,80] focused on improving breastfeeding, 26% (10/38) [44,46,47,49,52,60,61,71,73,81] focused on multiple behaviors, 16% (6/38) [50,54,66,74,75,78] on diet only, 5% (2/38) [63,68] on sleep only, and 3% (1/38) [57] on physical activity only. The multiple behavior interventions targeted different combinations of breastfeeding, feeding practices, diet, physical activity, sedentary behavior, screen time, and sleep. Only one study focused on all behaviors (breastfeeding, feeding practices, physical activity, sedentary behavior, screen time, and sleep) [61], and no study focused solely on sedentary behavior/screen time. One study included children aged 0‐3 years but is reported under the infant age group because the mean child age fell within infancy [72].

**Table 1.** Overview of the 38 randomized controlled trials of autonomously delivered digital interventions for healthy parent-child populations (0‐5 years), conducted across multiple countries and published up to January 2026.

Age group	Breastfeeding	Combined^a^	Diet	Physical activity	Sedentary behavior	Sleep
Newborn/infants^b^^,c^	19	2	2	0	0	1
Toddlers^d^	0	3	2	0	0	1
Preschoolers^e^	0	5	2	1	0	0

a The combined interventions focused on multiple behaviors eg, diet and physical activity/sedentary behavior.

b One of the studies targeted fathers; the others targeted mothers.

c 0-2 months of age for newborns and 3-11 months of age for infants.

d 12-35 months of age (≥1 to < 3 years).

e 36-59 months of age (≥3 to < 5 years).

Tables 2-4 present the study characteristics for the included studies divided by age group. In summary, the included studies comprised RCTs (n=32) [44-63,68,70-80], pilot RCTs (n=4) [47,64-66], one feasibility RCT [67], and one pilot study using a micro-RCT design [81]. Studies were published between 2011 and 2026, with most studies being published since 2020 (27/38, 71%) [51-62,64,65,69-81]. Most studies were conducted in the United States (n=14) [44,45,47,48,57,64-70,76], followed by Australia (n=5) [49,52,53,71,80], China (n=3) [51,61,72], Sweden (n=2) [46,60], Norway (n=2) [50,54], Ethiopia (n=2) [77,79], and Thailand (n=2) [73,74]. Single studies were conducted in India [56], Myanmar [59], Nepal [78], Iran [75], Turkey [58], Vietnam [55], Spain [62] and Canada [81]. The studies included sample sizes ranging from 18 [81] to 5095 [56].

**Table 2.** Risk of bias assessment table (using the Joanna Briggs Institute [JBI] Critical Appraisal Checklist for randomized controlled trials [41]).

Author (date)	Selection and allocation			Administration of intervention/exposure			Assessment, detection, and measurement of the outcome			Participant retention	Statistical conclusion validity		
	True randomization	Group allocation concealed	Groups similar at baseline	Participants blinded	Treatment deliverers blinded^a^	Groups treated identically (other than intervention)	Outcome assessors blinded	Outcomes measured the same way for groups	Outcomes measured in a reliable way	Follow-up complete	Intention-to-treat (ITT) analysis	Appropriate statistical analysis	Trial design appropriate
Pregnancy and infancy													
Breastfeeding													
Ahmed et al (2016) [45]	Y^b^	U^c^	Y	N^d^	N	Y	U	Y	Y	Y	Y	Y	Y
Unger et al (2018) [48]	Y	Y	N	N	N	Y	Y	Y	Y	Y	Y	Y	Y
Wu et al (2020) [51]	Y	Y	N	U	N	Y	Y	Y	U	Y	Y	Y	Y
Lewkowitz et al (2020) [69]	Y	Y	Y	Y	—^e^	Y	Y	Y	Y	Y	Y	Y	Y
Scott et al (2021) [53]	Y	N	Y	N	N	Y	U	Y	Y	Y	Y	Y	Y
Saucedo Baza et al (2022) [65]	Y	U	Y	N	N	Y	U	Y	Y	N	Y	Y	Y
Doan et al (2022) [55]	Y	Y	Y	Y	—	Y	Y	Y	Y	Y	Y	Y	Y
LeFevre et al (2022) [56]	Y	Y	Y	N	N	Y	Y	Y	U	Y	Y	Y	Y
Acar and Sahin (2023) [58]	Y	U	Y	N	U	Y	U	Y	U	Y	Y	Y	Y
Hmone et al (2023) [59]	Y	Y	N	N	U	Y	Y	Y	U	Y	Y	Y	Y
Vila-Candel et al (2024) [62]	Y	Y	N	U	N	N	Y	Y	U	Y	Y	Y	Y
Henshaw et al (2024) [76]	Y	U	U	N	—	Y	U	Y	Y	Y	N	Y	Y
De Mello et al (2025) [70]	Y	U	Y	N	N	Y	U	Y	U	N	N	Y	Y
Brown et al (2025) [80]	Y	Y	Y	Y	—	Y	U	Y	Y	U	Y	Y	Y
Cherie et al (2025) [79]	Y	U	Y	N	—	Y	Y	Y	Y	Y	Y	Y	Y
Gilano et al (2025) [77]	Y	U	Y	N	—	Y	Y	Y	Y	Y	Y	Y	Y
Breastfeeding and feeding practices													
Palacios et al (2018) [67]	Y	U	Y	U	—	Y	U	Y	Y	Y	Y	Y	Y
Davis et al (2023) [64]	Y	Y	N	N	N	Y	Y	Y	U	Y	Y	Y	Y
Li et al (2024) [72]^f^	Y	U	N	N	N	Y	U	Y	Y	N	Y	Y	Y
Combined^g^													
Wen et al (2020) [52]	Y	U	Y	N	—	Y	Y	Y	Y	Y	Y	Y	Y
Wu et al (2023) [61]	Y	N	N	N	N	N	N	Y	U	Y	Y	Y	Y
Diet													
Røed et al (2021) [54]	Y	U	Y	N	N	Y	U	Y	Y	Y	Y	Y	Y
Helle et al (2019) [50]	Y	Y	Y	N	—	Y	U	Y	Y	Y	Y	Y	Y
Sleep													
Moon et al (2017) [63]	Y	U	N	U	U	Y	U	Y	U	Y	U	Y	Y
Toddlerhood													
Combined^g^													
Alexandrou et al (2023) [60]	Y	N	Y	N	—	Y	Y	Y	Y	Y	Y	Y	Y
Sandborg et al (2025) [71]	Y	Y	N	N	U	Y	N	Y	Y	N	Y	Y	Y
Jongpaiboonpatana et al (2025) [73]	Y	U	Y	N	U	Y	Y	Y	U	Y	Y	Y	Y
Diet													
Cunningham et al (2023) [78]	Y	Y	Y	N	U	Y	Y	Y	Y	Y	Y	Y	Y
Hunsrisakhun et al (2025) [74]	Y	Y	Y	N	U	Y	Y	Y	Y	Y	N	Y	Y
Sleep													
Mindell et al (2011) [68]	U	U	N	Y	—	Y	Y	Y	Y	U	U	N	Y
Preschool													
Combined^g^													
Knowlden et al (2015) [44]	Y	U	Y	Y	U	N	Y	Y	Y	Y	Y	Y	Y
Delisle-Nyström et al (2017) [46]	Y	Y	Y	N	N	Y	Y	Y	Y	Y	Y	Y	Y
Sun et al (2017) [47]	U	U	Y	N	N	Y	Y	Y	Y	Y	Y	Y	Y
Hammersley et al (2019) [49]	Y	Y	Y	Y	N	N	Y	Y	Y	Y	Y	Y	Y
Diet													
Bakirci-Taylor et al (2019) [66]	Y	Y	N	U	N	Y	N	Y	Y	Y	N	Y	Y
Hojati et al (2024) [75]	Y	Y	Y	N	N	Y	Y	Y	Y	Y	U	Y	Y
Physical activity													
Staiano et al (2022) [57]	Y	U	Y	N	—	N	Y	Y	Y	Y	Y	Y	Y
Phillips et al (2026) [81]	Y	U	U	N	U	Y	U	Y	Y	N	N	Y	Y

a Due to the nature of the interventions under investigation (they were all autonomously delivered), blinding of treatment deliverers was not applicable in this context.

b Y: yes.

c U: unclear.

d N: no.

e Not applicable.

f This study enrolled children up to 3 years, but the average age in both arms fell within infancy; the study is therefore reported under the pregnancy-infancy category.

g The combined interventions focused on multiple behaviors eg, diet and physical activity/sedentary behavior.

**Table 3.** Study characteristics and effectiveness of autonomously delivered digital interventions focusing on pregnancy to infancy (0‐11 months; 0‐2 months of age for newborns and 3‐11 months of age for infants): randomized controlled trials published between 2011‐2026 and across multiple countries (n=24).

Author (date)	Country	N	Target group	Duration^a^	Follow up	Intervention type	Assessment	Breast-feeding	Feeding practices	Diet	PA^b^	SB^c^	ST^d^	Sleep
Breastfeeding														
Ahmed et al (2016) [45]	United States	141	Mother-newborn dyads	30 days	Hospital discharge, 1, 2, and 3 months post partum	Web-based	Survey	S/NS^e^	—^f^	—	—	—	—	—
Unger et al (2018) [48]	United States	300	Pregnant women (<36 wk pregnant)	Pregnancy (26 wk) to 12 weeks post partum	16 and 24 weeks post partum	SMS text messaging	Survey	S^g^	—	—	—	—	—	—
Wu et al (2020) [51]	China	344	Pregnant women (11‐37 wk pregnant)	Third month of pregnancy to 6 months post partum	BL^h^, 0‐1 month, 2‐3 months, and 4‐5 months post partum	WeChat (Tencent)	Interview	S/NS	S/NS^i^	—	—	—	—	—
Lewkowitz et al (2020) [69]	United States	170	Low-income first-time mothers (~36 wk pregnant)	Pregnancy (36 wk) to 6 weeks post partum	BL, postpartum day 2, 6 weeks, 3 and 6 months	App	Survey	NS^j^	—	—	—	—	—	—
Scott et al (2021) [53]	Australia	1426	Fathers	From recruitment in pregnancy until 6 months post partum	BL, 6 - and 26 weeks post partum	App	Survey	NS	NS^i^	—	—	—	—	—
Saucedo Baza et al (2022) [65]^l^	United States	36	Pregnant women (beyond 37 wk pregnant)	6 weeks	BL, 4‐6 wk post partum	App	Survey	NS	—	—	—	—	—	—
Doan et al (2022) [55]	Vietnam	568	Mothers who delivered by a cesarian section	Pregnancy to 4 months post partum	BL, 1-, 4- and 6-months	App	Interview	S/NS	—	—	—	—	—	—
LeFevre et al (2022) [56]	India	5095	Hindi speaking women (4‐7 mo pregnant)	Pregnancy (gestational wk 12‐34) to 12 months post partum	BL, 12 months	Voice calls	Survey	NS	—	—	—	—	—	—
Acar and Sahin (2023) [58]	Turkey	73	Primiparous mothers	8 weeks (First day to 8 wk post partum)	BL, 4, and 8 weeks	App	Survey	S^g^	—	—	—	—	—	—
Hmone et al (2023) [59]	Myanmar	353	Pregnant women (28 and 34 wk pregnant)	6 months (from gestational wk 28‐34 to 6 months of age)	BL, 1‐6 months	SMS text messaging	Interview	S	S	—	—	—	—	—
Vila-Candel et al (2024) [62]	Spain	270	Pregnant women (third trimester)	6 months (third trimester to 6 months of age)	Hospital discharge after delivery, 15 days, 6 weeks, 3, and 6 months	App	Survey	NS	—	—	—	—	—	—
Henshaw et al (2024) [76]	United States	128	Mother and partner	6 weeks	BL, 6 weeks, 6 months	Tablet-based program	Survey	NS	S/NS	—	—	—	—	—
De Mello et al (2025) [70]	United States	36	Pregnant women (32‐36 wk gestation)	Antenatal to 12 months after birth	BL (32‐36 wk gestation), 12 months	App	Survey	NS	NS	—	—	—	—	—
Brown et al (2025) [80]	Australia	5783	Mothers 1‐4 wk post partum	24 months	BL, 3 weeks, 3 months, 6 months, 9 months, 1 year, 18 months	SMS text messaging	Survey	NS	NS	—	—	—	—	—
Cherie et al (2025) [79]	Ethiopia	743	Pregnant women (26‐28 wk gestation)	Antenatal to 42 days post partum	BL, 60 days	SMS text messaging	Survey	U^k^	U	—	—	—	—	—
Gilano et al (2025) [77]	Ethopia	675	Pregnant women (16‐28 wk gestation)	Antenatal to 6 months after birth	BL, 1 month, 6 months	SMS text messaging	Interview	S	S	—	—	—	—	—
Breastfeeding and feeding practices														
Palacios et al (2018) [67]^l^	United States	202	Caregivers of infants 0‐2 months old participating in the WIC program, Intervention: infant age 0.93 (SD 0.44) months; Control: infant age 0.98 (SD 0.47) months	4 months	BL, 4 months	SMS text messaging	Survey	NS	NS	—	—	—	—	—
Davis et al (2023) [64]^i^	United States	38	Parent of an infant aged 3‐30 days	12 months	BL (0‐2 wk), 2‐4 months, 6‐9 mo, and 12 months	SMS text messaging	Survey	NS	NS	—	—	—	—	—
Li et al (2024) [72]^m^	China	1332	Caregivers of children 0‐3 years, Intervention: 8.6 (SD 7.2) months; Control: 9.0 (SD 6.8) months	9 months	BL and 9 months	WeChat	Survey	S/NS	S/NS	NS	—	—	—	—
Combined^n^														
Wen et al (2020) [52]	Australia	1155	Pregnant women (24‐34 wk pregnant)	Antenatal to 10 months after birth	BL, 6, and 12 months of child age	SMS text messaging	Survey	NS	S	—	NS	—	S	—
Wu et al (2023) [61]	China	1610	Infants and young children aged 6‐20 months old and their primary caregivers, 36% aged 6‐11 months, 64% aged 12‐20 months	2 months	BL, 1 month, and 2 months	WeChat	Survey	NS	S	S	S	NS	S	NS
Diet														
Røed et al (2021) [54]	Norway	291	Parents of infants and toddlers, mean age 10.9 (1.2) months old	6 months	BL and 6 months	Website	Survey	—	—	S/NS	—	—	—	—
Helle et al (2019) [50]	Norway	715	Mother of a 3‐5 month-old infant	12 months	BL (child age: 5 months), 12 months (intervention completion), and one year after the intervention (child age: 24 months)	Website	Survey	—	S/NS	NS	—	—	—	—
Sleep														
Moon et al (2017) [63]	United States	1600	Mothers of infants, mean age 11.2 (SD 4.4) weeks	60 days	BL and infant age 60 days	Email or SMS text messaging	Survey	—	—	—	—	—	—	S

a Due to the nature of recruitment in pregnancy, the duration of the intervention was not always clear.

b PA: physical activity.

c SB: sedentary behavior.

d ST: screen time.

e S/NS: some significant and some nonsignificant results.

f Not applicable,

g S: significant.

h BL: baseline.

i These interventions focused only on breastfeeding, but other infant feeding practice outcomes (eg, introduction to formula and complementary foods, giving dairy or dairy products, water, semisolid, or solid foods) were also assessed.

j NS: not significant.

k U: percentages reported for each group but insufficient information to determine statistical significance.

l These studies were pilot randomized controlled trials (RCT) or feasibility RCT.

m This study enrolled children up to 3 years, but the average age in both arms fell within infancy; the study is therefore reported under the pregnancy-infancy category.

n The combined interventions focused on multiple behaviors eg, diet and physical activity/sedentary behavior.

**Table 4.** Study characteristics and effectiveness of autonomously delivered digital interventions focusing on toddlers (12‐35 months; ≥1 to -<3 years): randomized controlled trials published between 2011‐2026 and across multiple countries (n=6).

Author (date)	Country	N	Target group	Duration	Follow-up	Intervention type	Assessment details	Feeding practices	Diet	PA^a^	SB^b^	ST^c^	Sleep
Combined^d^													
Alexandrou et al (2023) [60]^e^	Sweden	552	Parents with a 2.5‐3 y-old child, 2.5 years (n=403), 3 years (n=149)	6 months	BL^f^ and 6 months	App	Survey	—	S^g^	NS^h^	-	S	—
Sandborg et al (2025) [71]	Australia	1165	Parents with a child 18‐35 months (mean age 27.1 [SD 4.1] months)	12 months	BL and 6 months	App +SMS text messaging	Survey	—	—	S	—	NS	S
Jongpaiboonpatana et al (2025) [73]	Thailand	112	Parents of children aged 12‐36 months; Intervention 23.3 (SD 6.2) months; Control: 22.5 (SD 7.0) months	6 weeks	BL, 6, and 10 weeks	Online videos	Survey + interview	—	—	S	—	NS	—
Diet													
Cunningham et al (2026) [78]	Nepal	2537	Families with children aged 12‐23 months	1000 days	BL and 1000 days	SMS text messaging	Survey	—	NS	—	—	—	—
Hunsrisakhun et al (2025) [74]	Thailand	303	Parents of a child 6‐42 months; Group 1: 23.4 (SD 9.9) months; Group 2: 24.0 (SD 10.6) months	6 months	BL, 3, and 6 months	Facebook Messenger Chatbot (Meta)	Survey	S/NS^i^	NS	—	—	—	—
Sleep													
Mindell et al (2011) [68]	United States	264	Mothers and their infant or toddler (ages 6‐36 months, mean age 19.4 [SD 8.9] months)	2 weeks	Days 8 (BL), 15, and 22	Website +emails	Survey	—	—	—	—	—	S

a PA: physical activity.

b SB: sedentary behavior.

c ST: screen time.

d The combined interventions focused on multiple behaviors eg, diet and physical activity/sedentary behavior.

e This study focused on both the toddler period and preschool age.

f BL: baseline.

g S: significant.

h NS: not significant.

i S/NS: some significant and some nonsignificant results.

### Risk of Bias in Studies

The susceptibility to bias of the included studies is presented in Table 2. Briefly, all studies measured outcomes the same way for groups and had an appropriate study design (n=38) [44-81]. Most studies used appropriate statistical analyses (n=37) [44-67,69-81], had true randomization (n=36) [44-46,48-67,69-81], had complete follow-up (or, if not, adequately described and analyzed differences between groups in terms of their follow-up; n=31) [44-64,66,67,69,73-79], used intention-to-treat analysis (n=30) [44-62,64,65,67,69,71-73,77-80], and treated groups identically (other than the intervention of interest; n=33) [45-48,50-56,58-60,63-81]. Findings were mixed for other risk of bias items; in particular, concealment of group allocation (yes: n=17 [46,48-51,55,56,59,62,64,66,69,71,74,75,78,80]; unclear: n=18 [44,45,47,52,54,57,58,63,65,67,68,70,72,73,76,77,79,81]; no: n=3 [53,60,61]), similarity of groups at baseline (yes=25 [44-47,49,50,52-58,60,65,67,69,70,73-75,77-80]; no=11 [48,51,59,61-64,66,68,71,72]; unclear=2 [76,81]), blinding of participants (yes=6 [44,49,55,68,69,80]; no=27 [45-48,50,52-54,56-61,64,65,70-79,81]; unclear=5 [51,62,63,66,67]), blinding of outcome assessors (yes=22 [44,46-49,51,52,55-57,59,60,62,64,68,69,73-75,77-79]; no=3 [61,66,71]; unclear=13 [45,50,53,54,58,63,65,67,70,72,76,80,81]), reliability of outcome measures (yes=28 [44-50,52-55,57,60,65-69,71,72,74-81]; unclear=10 [51,56,58,59,61-64,70-73]). The item assessing the blinding of treatment deliverers was not applicable for many studies, as the digital interventions under investigation were all autonomously delivered.

### Results of Synthesis

#### Intervention Characteristics

Key intervention characteristics of the included studies spanning pregnancy to infancy, toddlers, and preschoolers are presented in Tables2^4^, respectively. Additional details on the intervention and control conditions for each age group are available (in Tables S2-S4 in Multimedia Appendix 2). Overall, intervention duration ranged from 2 weeks [68] to 1000 days [78]. The included studies used various delivery channels of digital technologies for the intervention. The most common digital tools used were apps (11/38, 29%) [46,53,55,57,58,60,62,65,69,70,75], followed by SMS text messaging (10/38, 26%) [48,52,59,64,67,77-81], and web- or internet-based (6/38, 16%) [44,45,49,50,54,66]. Other digital tools used included WeChat (Tencent; 3/38, 8%) [51,61,72], online videos (1/38, 3%) [73], a combination of app and SMS text messaging (1/38, 3%) [71], websites and emails (1/38, 3%) [68], or email and SMS text messaging (1/38, 3%) [63], a tablet-based program (2/38, 5%) [47,76], automated voice calls (1/38, 3%) [56], and Facebook Messenger Chatbot (Meta; 1/38, 3%) [74].

#### Assessment of Target Outcomes

As shown in Tables 2-4, a broad range of methods and definitions of outcomes were used, with varying numbers as well as timing of the follow-up measures. Outcomes were mainly assessed by parent report using a range of different questionnaires, except for 5 studies where objective methods were used. Four studies [46,49,57,81] used accelerometry to assess physical activity and sedentary behavior, and one study [66] used reflective spectroscopy to assess dietary intake (fruit and vegetable consumption).

#### Intervention Effectiveness

##### Effectiveness of Studies Focusing on Pregnancy to Infancy

The level of effectiveness of the studies spanning pregnancy to infancy is presented in Table 3 (overview) and Table S5 in Multimedia Appendix 2 (details).

Among the studies that targeted breastfeeding, most reported no difference in breastfeeding outcomes between the groups [53,56,62,65,69,70,80], or mixed results [45,51,55,72,76], while one study [79] reported breastfeeding outcomes as percentages only, with insufficient information to determine statistical significance. Of the interventions that showed an effect on one or more of the breastfeeding outcomes [45,48,51,55,58,59,76,77], most targeted mothers already in pregnancy, ranging from gestational week 11-37 [48,51,55,59,77] with the remainder commencing post-birth [45,58,76] (Table 3 and Table S5 in Multimedia Appendix 2). The duration of the successful interventions ranged between 30 days and up to 6 months post partum, with a variety in digital delivery modes including an app [55,58], SMS text messaging [48,59,77], web-based [45], tablet-based [76], and WeChat [51] (Table S2 in Multimedia Appendix 2).

Three of the studies also reported results for the effectiveness of the intervention on feeding practices, showing a significant reduction in bottle-feeding at 6 months in the intervention group [59] but no difference in the introduction of formula and complementary foods between the groups [53]. One study reported mixed results with a significantly lower rate of giving dairy products to the child 0‐1 months post partum in the intervention groups but no difference in giving semisolid or solid foods at 0‐1 month, 2‐3 months, and 4‐5 months [51]. Three studies targeted both breastfeeding and feeding practices, with most reporting nonsignificant [64,67] or mixed results [72]. In addition, the studies targeting a combination of behaviors [52,61] reported significant improvements in feeding-related outcomes (eg, appropriate timing of solid foods and diet diversity).

The 2 studies targeting diet during infancy reported mixed findings [50,54]. In more detail, Røed et al [54] reported a larger increase in the frequency of vegetable intake in the intervention group compared to the control group but no difference in child food intake of fruits, vegetables, and discretionary foods between baseline and 6 months. In contrast, Helle et al [50] reported nonsignificant findings for all dietary intake variables, child mealtime habits, frequency of family meals, maternal feeding practices, and maternal feeding style at 12 months. However, impact was reported for other feeding practices, with significantly higher food responsiveness and lower emotional overeating in the intervention group compared to the control [50]. Both interventions were delivered via a website and included intervention content in video as well as recipes. Røed et al [54] also included modules with lessons, activity elements (eg, quizzes), a discussion forum, and weekly emails was half as long as that of Helle et al [50] (6 months vs 12 months), and targeted older infants (mean age 11 months vs 3‐5 months old).

Only one study targeted sleep in the infancy period and reported significant improvements for infant sleep practices in the intervention group [63]. Sleep practices included a higher prevalence of placing their infant in a supine position, room sharing without bed sharing, no soft bedding use, and any pacifier use. The intervention was 60 days and included health messages and educational videos delivered by email or SMS text messages.

##### Effectiveness of Studies Focusing on Toddlers

The effectiveness of studies focusing on toddlers is presented in Table 4 (overview) and Table S6 in Multimedia Appendix 2 (details). Among the interventions targeting multiple behaviors, one reported significant improvements in diet (higher vegetable intake and lower intakes of sweet and savory treats and sweet drinks) and less screen time but no differences in physical activity [60]. The other studies reported significant improvements in parental knowledge of child movement behaviors and sleep [71] and increases in parent-child play frequency [73], but no significant improvements related to screen time [71,73].

The 2 studies targeting diet also reported mixed findings [74,78]. One intervention did not demonstrate overall improvements in dietary diversity [78]. The other intervention reported several significant improvements in feeding-related behaviors (eg, reduced bottle use and fewer night feedings) but also nonsignificant outcomes (eg, consuming sweet foods) [74].

The other study in this age group targeted sleep only and reported improved sleep (decreased sleep onset latency, decreased number/duration of night wakings, increased sleep continuity, and increased nighttime sleep) in the intervention group compared to the control group [68]. The intervention duration and digital delivery mode varied; one was 6 months in duration and delivered using an app [60], while the sleep intervention was 2 weeks and delivered via a website and emails [68] (Table 4 and Table S3 in Multimedia Appendix 2).

##### Effectiveness of Studies Focusing on Preschoolers

The effectiveness of the studies focusing on preschoolers is presented in Table 5 (overview) and Table S7 in Multimedia Appendix 2; details). Among the studies targeting a combination of behaviors, most reported significant improvements for feeding practices (2/2, 100%) [47,49] and diet (3/4, 75%) [44,46,49], for example, lower intakes of sweetened beverages [44,46] and discretionary foods [49], and higher intakes of fruit and vegetables [44], while one study reported null effects for child eating style and eating related to hunger [47]. All studies targeting a combination of behaviors reported null effects for physical activity (3/3, 100%) [46,47,49], sedentary behavior (2/2, 100%) [44,46], and sleep (1/1, 100%) [49]. The 2 studies targeting screen time reported both positive [44] and null results [49]. Similarly, the studies targeting physical activity reported no overall effects on physical activity or sedentary behavior [57,81], except for context-specific effects (eg, presence of a parent, weather-dependent) and increased parental moderate-to-vigorous physical activity [81].

**Table 5.** Study characteristics and effectiveness of autonomously delivered digital interventions focusing on the preschool age (36‐59 months; ≥3 to -<5 y): randomized controlled trials published between 2011 and 2026 and across multiple countries (n=8).

Author (date)	Country	N	Target group	Duration	Follow up	Digital tool	Assessment details	Feeding practices	Diet	PA^a^	SB^b^	ST^c^	Sleep
Combined^d^													
Knowlden et al (2015) [44]	United States	57	Mothers of 4‐6-year-old children	8 weeks	BL^e^, 4, and 8 weeks	Website	Survey	—^f^	S	—	NS^g^	S^h^	—
Delisle-Nyström et al (2017) [46]	Sweden	315	Parents of children aged 4 years, Intervention: 4.5 (SD 0.1) years, 45% female, maternal age 36.0 (SD 4.1) years; Control: 4.5 (SD 0.1) years	6 months	BL, 6 months	App	Direct observation (photos) and acceler-ometry	—	S	NS	NS	—	—
Sun et al. (2017) [47]^i^	United States	32	Chinese mothers and their children 3‐5-year-old child, mean age 4.3 (SD 0.7) years	8 weeks	BL, 3, and 6 months	Tablet-based program	Survey	S	NS	NS	—	—	—
Hammersley et al (2019) [49]	Australia	86	Parents of 2‐5 y-old children, mean age 3.5 (SD 0.9) years	11 weeks	BL, 3, and 6 months	Website	Survey and acceler-ometry	S	S	NS	—	NS	NS
Diet													
Bakirci-Taylor et al. (2019) [66]^i^	United States	30	Parents and children aged 3‐8 years, Intervention: child age 3.8 (SD 0.8) years; Control: child age 3.6 (SD 1.4) years	10 weeks	BL, 5- and 10 weeks	Website	Survey, direct observation (photos) and reflective spectroscopy	—	S/NS^j^	—	—	—	—
Hojati et al (2024) [75]	Iran	116	Mothers and children aged 2‐6 y with confirmed undernutrition, Intervention: 50.3 (SD 11.6) months; Control: 46.51 (SD 13.3) months	3 months	BL, 3 months	App	Survey	S	—	—	—	—	—
Physical activity													
Staiano et al (2022) [57]	United States	72	Parents and 3‐5-year-old children, mean age 4.0 (SD 0.8) years	12 weeks	BL, 12 weeks (end of intervention), and 24-week (follow-up)	App	Direct observation and acceler-ometry	—	—	NS	NS	—	—
Phillips et al (2026) [81]	Canada	18	Parents and children aged 3‐4 years	2 weeks	2 weeks; 7 prompts/day; 60-min postprompt outcome windows	SMS text messaging	Acceler-ometry	—	—	S/NS	NS	—	—

a PA: physical activity.

b SB: sedentary behavior.

c ST: screen time.

d The combined interventions focused on multiple behaviors eg, diet and physical activity/sedentary behavior.

e BL: baseline

f Not applicable.

g NS: not significant.

h S: significant.

i These studies were pilot randomized controlled trials.

j S/NS: some significant and some nonsignificant results.

The studies targeting diet only reported higher vegetable intake in the intervention group but no difference in the frequency of fruit and vegetable consumption [66] and significant improvements in maternal nutrition knowledge, feeding attitudes, and nutrition practices [75].

The interventions with an effect on diet and/or feeding practices ranged from 8 weeks to 6 months, with the majority being 8‐11 weeks [44,47,49,66,75], and the digital delivery mode included using a website or web-based intervention [44,49,66], an app [46,75] and a tablet-based program [47] (Table 5 and Table S4 in Multimedia Appendix 2). The study that had a significant intervention effect on screen time targeted 4‐6-year-olds (n=57) and delivered a web-based intervention over 8 weeks [44]. Hammersley et al [49] evaluated the effectiveness of a website but found no effect on screen time. Their intervention duration was longer (11 wk), and the participating children were younger (mean age ~3.5, SD 0.9 years; n=86).

### Intervention Development and Engagement

Co-design, intervention theory, process evaluation, and engagement outcomes for studies focusing on pregnancy to infancy, toddlers, and preschoolers are shown in Tables S8, S9, and S10 in Multimedia Appendix 2, respectively.

#### Co-Design

Of the 38 interventions, 24 (63%) [45-49,51,53-56,58-61,63,64,67,69,72,74,75,78-80] reported some form of co-design or end-user engagement in the development of the intervention. The extent of co-design or end-user engagement ranged from end users’ (ie, parents’) views on delivery platforms [45] and pilot testing content with end users (eg, [46]) to intensive qualitative work involving a range of stakeholders to inform all aspects of the intervention [63]. Of the 24 interventions reporting some form of co-design, 16 (67%) showed some evidence of effectiveness. By comparison, 10 of the 14 interventions (71%) that did not report any form of co-design showed some evidence of effectiveness.

#### Intervention Theory

Twenty-seven of the 38 interventions (71%) [44-47,49,50,52-55,57,59,60,64-67,70-74,76-78,80,81] reported being underpinned by theory. The most commonly used approaches were social-cognitive and learning-based theories, including Social Cognitive Theory [44-46,49,50,53,54,57,60,65,66], the Health Belief Model [52,59,64], social learning theory [73], Protection Motivation Theory [74], and behavior-change communication frameworks [77]. Motivational and self-regulatory models were also used, such as Self-Determination Theory [81], the TransTheoretical model of health behavior change [67], the Information Motivation Behavioral Skills model [47], and breastfeeding self-efficacy frameworks [70,76]. Several interventions used comprehensive design frameworks, including the Behavior Change Wheel [71,80], the Theoretical Domains Framework [80], and the World Health Organization (WHO) comprehensive health literacy model [72], while others used technology-oriented or implementation-focused models such as the Behavioral Intervention Technology Model [55], AI-supported behavior-change models [74], and a formal theory of change [78]. Of the 27 interventions that reported being underpinned by theory, 19 (70%) showed some evidence of effectiveness. By comparison, 6 of the 11 (55%) interventions that did not report any use of theory to underpin the intervention showed some evidence of effect.

#### Process Evaluation

Process evaluation results were reported for 24 studies (63%). These mostly focused on subjective reports of acceptability, satisfaction, and usefulness (n=17) [44,45,47,49,53,57,58,60,61,65,66,68-70,74,80,81] and/or objective measures of delivery/use (eg, successful delivery of SMS text messaging, usage data for websites/apps; n=9) [46,48,51-54,64,67,69]. Interventions using mobile apps were most consistently found to be acceptable and useful; apps were predominantly rated highly for ease of use, design, and helpfulness [46,53,57,58,69]. Although SMS text messaging interventions were generally found to be acceptable and useful, some studies reported declining engagement over time [52] or technical issues (eg, issues with SMS text messaging delivery [67] or phone service [64]). Web-based interventions showed mixed results in terms of acceptability and usage, with studies generally finding lower engagement compared to apps; for example, Røed et al [54] reported that 13% of participants never used the website, while Bakirci-Taylor et al [66] reported that participants engaged more with the Facebook page than the mobile website. Other interventions using Facebook reported less positive results; for example, Hammersley et al [49] reported that only 39% of participants found the Facebook component useful. In contrast, the Facebook Messenger Chatbot intervention reported high satisfaction (mean score 4.0 out of 5; SD 0.5-0.6 across groups) [74].

#### Engagement

Engagement outcomes were reported by 24 studies (63%); n=7 [50,57,58,61,65,70,78] used subjective measures (eg, self-reported use), n=13 [46,48,52,54,56,62,63,66,67,69,71,72,74] used objective measures (eg, app analytics), and n=4 [49,53,60,80] used both. Of these, 6 studies examined the impact of engagement on intervention effectiveness, with mixed findings across behaviors. All breastfeeding-focused interventions reported no impact on the outcomes [56,62,69], whereas studies targeting breastfeeding and feeding practices [72], diet [78], and movement behaviors [71] found that higher engagement (eg, more videos viewed, messages opened, or greater app use) was associated with more favorable outcomes.

## Discussion

### Principal Findings

This systematic review provides a comprehensive overview of autonomously delivered digital interventions targeting multiple behaviors in the first 2000 days and examines co-design, engagement, and process evaluation, areas rarely assessed in previous reviews. Thirty-eight interventions were included in the review, with most focusing on improving breastfeeding practices by targeting mothers and their youngest children (newborns and infants) and a growing number of studies targeting toddlers and preschoolers. Overall, intervention designs varied considerably, and results were mixed for all targeted age groups with no apparent trend in intervention characteristics (eg, target behavior and digital delivery mode) for interventions shown to be effective. Although most studies reported some form of co-design or end-user engagement, very few examined the impact of engagement on the efficacy of the intervention.

The variability in intervention design is not unique for autonomously delivered digital interventions, and previous reviews on digital interventions for improving breastfeeding have similarly reported heterogeneity; for example, in delivery modes [82-84]. In terms of effectiveness, most of the included studies in our review reported no differences in breastfeeding outcomes between the groups and reported mixed results. These findings are similar to previous systematic reviews and meta-analyses focusing solely on mobile apps [83], remote provision of breastfeeding support education (eg, telephone, SMS text messaging, social media, video call, and email) [82], or mHealth-based interventions to promote breastfeeding [84]. To illustrate, Ziebart et al [83] found insufficient evidence for sustained beneficial effects of breastfeeding promotion and support through mobile apps on breastfeeding rates, while Gavine et al [82] concluded that remote interventions can be effective for improving exclusive breastfeeding at 3 months but with low certainty of evidence due to risk of bias, substantial heterogeneity, and imprecision in some outcomes. In contrast, Qian et al [84] reported, amongst other things, improvements in exclusive breastfeeding rates up to 6 months after delivery in comparison with usual care. These previous reviews included only up to 4 of the studies captured in our review, likely due to differences in search timeframes and inclusion/exclusion criteria. For example, Ziebart et al [83] focused exclusively on mobile apps related to breastfeeding and excluded web-based interventions; Gavine et al [82] limited their search to studies published after 2010 and focused on remote care broadly; and Qian et al [84] included a wider range of mHealth modalities (eg, phone calls, SMS text messaging, and interactive systems) but only considered breastfeeding outcomes. In contrast, our review included RCTs targeting a broader set of health behaviors (breastfeeding, feeding practices, diet, physical activity, sedentary behavior, and sleep) in children aged 0‐5 years, delivered solely via autonomous digital technologies. Thus, our review provides an important addition to the existing evidence.

Moreover, we report the effectiveness of interventions covering the first 5 years of life, which is recognized as a critical period for establishment of health behaviors. The studies targeting toddlers showed promising results for diet, screen time, and sleep but not physical activity. However, there were only 6 [60,68,71,73,74,78] studies in this age group, highlighting the need for more research targeting toddlers. Similarly, the number of studies targeting preschoolers was limited, and results indicated significant improvements for feeding practices and diet, mixed findings for screen time, and no overall differences for children’s physical activity, sedentary behavior, and sleep, although one trial reported context-specific reductions in children’s sedentary time and increased parental moderate-to-vigorous physical activity. Previous systematic reviews on digital interventions to promote these health behaviors in preschoolers have also reported mixed findings [25,26]. For example, Zhou et al [26] reported significant improvements for dietary behaviors and sleep but no significant improvements in physical activity for parent-based eHealth interventions. In contrast, a systematic review and meta-analysis on the effectiveness of eHealth interventions for promoting 24-hour movement behaviors in preschoolers [25] reported small but positive effects on physical activity, sedentary time, and sleep. One potential explanation for the discrepancies in results could be differences in intervention delivery, as most of the included studies in the meta-analysis relied on human interaction (eg, courses with staff members, motivational coaching, individual discussion/telephone calls, face-to-face workshop, and home visits) [25,26]. Although autonomously delivered interventions have greater potential for scalability and potential cost-effectiveness, digital interventions including a delivery personnel component might be more effective; however, considering the paucity of studies in this age group, more research is required to determine this.

Reporting of elements related to the design and evaluation of interventions, including co-design and process evaluation, is important considering that these can help improve intervention effectiveness [85] and identify key components that contribute to the success of interventions [86], respectively. Another important aspect is participant engagement, as it can provide information on levels of intervention exposure and uptake, which are crucial for effectiveness, as well as help identify factors that may optimize engagement [33] and ultimately inform the development of more impactful interventions. Although most of the included studies reported some form of co-design or end-user engagement, only 6 studies [56,62,69,71,72,78] examined the impact of engagement on intervention effectiveness. In contrast with previous findings in other populations (eg, [87,88]) and outcomes such as parenting practices and cognitions [71], the studies included in this review reported mixed findings across behaviors. All breastfeeding-focused interventions reported that higher engagement was not associated with intervention effectiveness on the targeted outcomes (ie, breastfeeding) [56,62,69], while studies targeting breastfeeding and feeding practices [72], diet [78], and movement behaviors [71] found that higher engagement was associated with more favorable outcomes. Nevertheless, the low reporting rate of engagement suggests that the results from this review about the impact may not fully reflect the intended interventions. Although engagement data are supposedly easy to collect in digital interventions (eg, apps), previous research has highlighted a gap in evaluating and reporting how engagement influences intervention effectiveness [89]. This raises critical questions, such as why engagement data are often omitted, and whether low engagement levels could be a contributing factor, with researchers hesitant to report underwhelming results. Despite the assumption that digital interventions increase reach, this may not translate to increased engagement [34]. Ultimately, understanding whether and how engagement mediates outcomes is essential to determine the true value of these interventions, and addressing these gaps is crucial for ensuring that scalable interventions are also impactful and meaningfully engaged with by users. This review highlights key considerations for improving future research, including the evaluation and reporting of the impact of engagement on the effectiveness of digital interventions for promoting health behaviors in early childhood.

### Strengths and Limitations

This review has several strengths and limitations. A key strength was the systematic approach used to search, screen, and synthesize the literature, including the PROSPERO registration of the review protocol and the use of the JBI Critical Appraisal Checklist for RCTs. Moreover, we focused on digital interventions that were delivered autonomously and thus, in theory, have the capacity to be scaled up and delivered at large without heavy researcher or staff input. Another strength is that we also considered intervention development (co-design and intervention theory) and participant engagement, which are important elements for intervention effectiveness [85]; however, this broader evaluation was constrained by the extent of reporting within individual studies. The present review also has limitations to acknowledge. First, despite comprehensive searches across multiple databases, it is possible that relevant studies were missed, particularly unpublished or non-English studies. Second, we restricted the review to autonomously delivered digital interventions, which limit our findings to hybrid models that include human support. Third, considerable heterogeneity in study populations, intervention content, delivery formats, and outcome measures prevented and limited our ability to perform a meta-analysis. The wide variety in intervention objectives, settings, methodologies, and delivery modes also made it difficult to compare findings across studies. Finally, digital health interventions evolve rapidly, and more recent innovations may not yet be represented in the current evidence base, as highlighted by recent evidence underscoring the challenges of synthesizing findings in this fast-moving field [22].

In terms of strengths and limitations for the individual studies, although most studies had an appropriate study design and use of statistical methods, findings were mixed for other risk of bias items, including concealment of group allocation, blinding of participants, and outcome assessors, as well as reliability of outcome measures. Considering the nature of the interventions, blinding of participants and researchers might not be feasible, suggesting that checklists assessing study quality specific to digital interventions are warranted. Most studies also used subjective methods to assess outcomes, which are inherently subject to misreporting biases. Finally, most studies were conducted in high-income countries, which limits generalizability to low-income countries.

### Conclusion

This review shows that autonomously delivered digital interventions for early childhood are highly heterogeneous and demonstrate mixed effectiveness, making it difficult to identify which components are most impactful. It is innovative in synthesizing evidence across the first 2000 days while simultaneously examining co-design, engagement, and implementation factors, dimensions rarely brought together in previous work. Unlike earlier reviews that focus on older children, single behaviors, or interventions involving human support, this review focuses solely on scalable autonomously delivered digital interventions for children 0‐5 years, a formative period for long-term health. Most importantly, it adds new insight by identifying three priority evidence gaps: (1) the scarcity of studies targeting toddlers and preschoolers, (2) inconsistent and incomplete reporting of engagement, and (3) limited understanding of how engagement influences outcomes. While autonomous digital interventions offer clear advantages in reach and scalability, their usefulness ultimately depends on whether interventions remain engaging, relevant, and effective for families. Together, these findings define priority areas for future research and clarify what is needed to strengthen the evidence base for scalable digital interventions in early childhood.

## Supplementary material

10.2196/85525 Multimedia Appendix 1 Full search strategy for all databases included in this systematic review, detailing search terms, Boolean operators, filters, and date limits used across Embase, Academic Search Complete, CINAHL Complete, Global Health, MEDLINE Complete, PsycINFO, and SPORTDiscus during searches conducted in December 2022, August 2024, and January 2026.

10.2196/85525 Multimedia Appendix 2 Detailed characteristics of included studies.

10.2196/85525 Checklist 1 PRISMA-S checklist.

## Abbreviations

JBI: Joanna Briggs Institute
mHealth: mobile health
PICOS: Population, Intervention, Comparison, Outcomes, and Study design
PRISMA: Preferred Reporting Items for Systematic Reviews and Meta-Analyses
PRISMA-S: Preferred Reporting Items for Systematic Reviews and Meta-Analyses Literature Search Extension
PROSPERO: International Prospective Register of Systematic Reviews
RCT: randomized controlled trial
WHO: World Health Organization

## References

[1] North K, Gao M, Allen G, Lee AC (2022). Breastfeeding in a global context: epidemiology, impact, and future directions. Clin Ther.

[2] Tapia-Serrano MA, Sevil-Serrano J, Sánchez-Miguel PA, López-Gil JF, Tremblay MS, García-Hermoso A (2022). Prevalence of meeting 24-Hour Movement Guidelines from pre-school to adolescence: a systematic review and meta-analysis including 387,437 participants and 23 countries. J Sport Health Sci.

[3] McArthur BA, Volkova V, Tomopoulos S, Madigan S (2022). Global prevalence of meeting screen time guidelines among children 5 years and younger: a systematic review and meta-analysis. JAMA Pediatr.

[4] Lioret S, Campbell KJ, McNaughton SA (2020). Lifestyle patterns begin in early childhood, persist and are socioeconomically patterned, confirming the importance of early life interventions. Nutrients.

[5] Craigie AM, Lake AA, Kelly SA, Adamson AJ, Mathers JC (2011). Tracking of obesity-related behaviours from childhood to adulthood: a systematic review. Maturitas.

[6] Cattan S, Fitzsimons E, Goodman A, Phimister A, Ploubidis GB, Wertz J (2024). Early childhood inequalities. Oxf Open Econ.

[7] Vicat-Blanc L, Merry L, Harguindéguy-Lincourt MC, Tang Y, Van Hulst A (2025). Co-design of interventions and services with structurally marginalized populations in the context of maternal and early childhood primary care: a rapid scoping review. Prim Health Care Res Dev.

[8] Gautam N, Dessie G, Rahman MM, Khanam R (2023). Socioeconomic status and health behavior in children and adolescents: a systematic literature review. Front Public Health.

[9] Brushe ME, Lynch JW, Melhuish E, Reilly S, Mittinty MN, Brinkman SA (2023). Objectively measured infant and toddler screen time: findings from a prospective study. SSM Popul Health.

[10] Nyberg G, Helgadóttir B, Moraeus L, Sipinen JP, Lindroos AK, Fröberg A (2026). A national sample of Swedish young children shows sociodemographic variations in physical activity and screen time. Acta Paediatr.

[11] Kracht CL, Webster EK, Staiano AE (2019). Sociodemographic differences in young children meeting 24-hour movement guidelines. J Phys Act Health.

[12] Askie LM, Espinoza D, Martin A (2020). Interventions commenced by early infancy to prevent childhood obesity-the EPOCH collaboration: an individual participant data prospective meta-analysis of four randomized controlled trials. Pediatr Obes.

[13] Blake-Lamb TL, Locks LM, Perkins ME, Woo Baidal JA, Cheng ER, Taveras EM (2016). Interventions for childhood obesity in the first 1,000 days a systematic review. Am J Prev Med.

[14] Hodder RK, O’Brien KM, Wyse RJ (2024). Interventions for increasing fruit and vegetable consumption in children aged five years and under. Cochrane Database Syst Rev.

[15] Mihrshahi S, Jawad D, Richards L (2021). A review of registered randomized controlled trials for the prevention of obesity in infancy. Int J Environ Res Public Health.

[16] Grady A, Jackson J, Wolfenden L, Lum M, Yoong SL (2023). Assessing the scalability of healthy eating interventions within the early childhood education and care setting: secondary analysis of a Cochrane systematic review. Public Health Nutr.

[17] Liu S, Ma J, Sun M, Zhang C, Gao Y, Xu J (2025). Mapping the landscape of digital health intervention strategies: 25-year synthesis. J Med Internet Res.

[18] Laws RA, Litterbach EKV, Denney-Wilson EA (2016). A comparison of recruitment methods for an mHealth intervention targeting mothers: lessons from the growing healthy program. J Med Internet Res.

[19] Brown V, Tran H, Downing KL, Hesketh KD, Moodie M (2021). A systematic review of economic evaluations of web-based or telephone-delivered interventions for preventing overweight and obesity and/or improving obesity-related behaviors. Obes Rev.

[20] Fiedler J, Eckert T, Wunsch K, Woll A (2020). Key facets to build up eHealth and mHealth interventions to enhance physical activity, sedentary behavior and nutrition in healthy subjects - an umbrella review. BMC Public Health.

[21] Alley SJ, Waters KM, Parker F (2024). The effectiveness of digital physical activity interventions in older adults: a systematic umbrella review and meta-meta-analysis. Int J Behav Nutr Phys Act.

[22] McDermott KT, Noake C, Wolff R (2023). Digital interventions to moderate physical inactivity and/or nutrition in young people: a cancer prevention Europe overview of systematic reviews. Front Digit Health.

[23] Hammersley ML, Jones RA, Okely AD (2016). Parent-focused childhood and adolescent overweight and obesity eHealth interventions: a systematic review and meta-analysis. J Med Internet Res.

[24] Bonvicini L, Pingani I, Venturelli F (2022). Effectiveness of mobile health interventions targeting parents to prevent and treat childhood obesity: systematic review. Prev Med Rep.

[25] Jiang S, Ng JYY, Chong KH, Peng B, Ha AS (2024). Effects of eHealth interventions on 24-hour movement behaviors among preschoolers: systematic review and meta-analysis. J Med Internet Res.

[26] Zhou P, Li Y, Lau PW, Yan L, Song H, Shi TL (2024). Effectiveness of parent-based electronic health (*eHealth*) intervention on physical activity, dietary behaviors, and sleep in preschoolers: a systematic review. J Exerc Sci Fit.

[27] Wang JW, Zhu Z, Shuling Z (2024). Effectiveness of mHealth app-based interventions for increasing physical activity and improving physical fitness in children and adolescents: systematic review and meta-analysis. JMIR Mhealth Uhealth.

[28] Baumann H, Fiedler J, Wunsch K, Woll A, Wollesen B (2022). mHealth interventions to reduce physical inactivity and sedentary behavior in children and adolescents: systematic review and meta-analysis of randomized controlled trials. JMIR Mhealth Uhealth.

[29] He Z, Wu H, Yu F (2021). Effects of smartphone-based interventions on physical activity in children and adolescents: systematic review and meta-analysis. JMIR Mhealth Uhealth.

[30] Singh B, Ahmed M, Staiano AE (2025). Lifestyle eHealth and mHealth interventions for children and adolescents: systematic umbrella review and meta-meta-analysis. J Med Internet Res.

[31] Ishaque S, Ela O, Dowling A (2025). Mobile health interventions for modifying indigenous maternal and child-health related behaviors: systematic review. J Med Internet Res.

[32] Yardley L, Spring BJ, Riper H (2016). Understanding and promoting effective engagement with digital behavior change interventions. Am J Prev Med.

[33] Schoeppe S, Alley S, Van Lippevelde W (2016). Efficacy of interventions that use apps to improve diet, physical activity and sedentary behaviour: a systematic review. Int J Behav Nutr Phys Act.

[34] Sandborg J, Markides BR, Simmons S (2025). Parental and demographic predictors of engagement in an mHealth intervention: observational study from the let’s grow trial. JMIR Mhealth Uhealth.

[35] National Health and Medical Research Council (2012). Infant feeding guidelines. https://www.nhmrc.gov.au/about-us/publications/infant-feeding-guidelines-information-health-workers.

[36] (2021). Recommendations for infants, toddlers and preschoolers (birth to 5 years). Australian Government Department of Health, Disability and Ageing.

[37] Markides B, Marshall S, Laws R, Hesketh K, Downing KL Systematic review of mHealth interventions targeting parents and caregivers to improve early childhood movement, nutrition and sleep behaviours. PROSPERO.

[38] Page MJ, McKenzie JE, Bossuyt PM (2021). The PRISMA 2020 statement: an updated guideline for reporting systematic reviews. BMJ.

[39] Rethlefsen ML, Kirtley S, Waffenschmidt S (2021). PRISMA-S: an extension to the PRISMA statement for reporting literature searches in systematic reviews. Syst Rev.

[40] Elicit: the AI research assistant. Elicit.

[41] Barker TH, Stone JC, Sears K (2023). The revised JBI critical appraisal tool for the assessment of risk of bias for randomized controlled trials. JBI Evid Synth.

[42] Deeks JJ, Higgins JP, Altman DG, McKenzie JE, Veroniki AA, Higgins JPT, Thomas J, Chandler J, Cumpston M, Li T, Page MJ, Welch VA (2024). Cochrane Handbook for Systematic Reviews of Interventions.

[43] Campbell M, McKenzie JE, Sowden A (2020). Synthesis without meta-analysis (SWiM) in systematic reviews: reporting guideline. BMJ.

[44] Knowlden AP, Sharma M, Cottrell RR, Wilson BRA, Johnson ML (2015). Impact evaluation of enabling mothers to prevent pediatric obesity through web-based education and reciprocal determinism (EMPOWER) randomized control trial. Health Educ Behav.

[45] Ahmed AH, Roumani AM, Szucs K, Zhang L, King D (2016). The effect of interactive web-based monitoring on breastfeeding exclusivity, intensity, and duration in healthy, term infants after hospital discharge. J Obstet Gynecol Neonatal Nurs.

[46] Nyström CD, Sandin S, Henriksson P (2017). Mobile-based intervention intended to stop obesity in preschool-aged children: the MINISTOP randomized controlled trial. Am J Clin Nutr.

[47] Sun A, Cheng J, Bui Q, Liang Y, Ng T, Chen JL (2017). Home-based and technology-centered childhood obesity prevention for Chinese mothers with preschool-aged children. J Transcult Nurs.

[48] Unger JA, Ronen K, Perrier T (2018). Short message service communication improves exclusive breastfeeding and early postpartum contraception in a low- to middle-income country setting: a randomised trial. BJOG.

[49] Hammersley ML, Okely AD, Batterham MJ, Jones RA (2019). An internet-based childhood obesity prevention program (Time2bHealthy) for parents of preschool-aged children: randomized controlled trial. J Med Internet Res.

[50] Helle C, Hillesund ER, Wills AK, Øverby NC (2019). Evaluation of an eHealth intervention aiming to promote healthy food habits from infancy -the Norwegian randomized controlled trial early food for future health. Int J Behav Nutr Phys Act.

[51] Wu Q, Huang Y, Liao Z, van Velthoven MH, Wang W, Zhang Y (2020). Effectiveness of WeChat for improving exclusive breastfeeding in Huzhu county China: randomized controlled trial. J Med Internet Res.

[52] Wen LM, Rissel C, Xu H (2020). Effects of telephone and short message service support on infant feeding practices, “Tummy Time,” and screen time at 6 and 12 months of child age: a 3-group randomized clinical trial. JAMA Pediatr.

[53] Scott JA, Burns SK, Hauck YL (2021). Impact of a face-to-face versus smartphone app versus combined breastfeeding intervention targeting fathers: randomized controlled trial. JMIR Pediatr Parent.

[54] Røed M, Medin AC, Vik FN (2021). Effect of a parent-focused eHealth intervention on children’s fruit, vegetable, and discretionary food intake (Food4toddlers): randomized controlled trial. J Med Internet Res.

[55] Doan TTD, Pham NM, Binns C (2022). Effect of a mobile application on breastfeeding rates among mothers who have cesarean deliveries: a randomized controlled trial. Breastfeed Med.

[56] LeFevre AE, Shah N, Scott K (2022). The impact of a direct to beneficiary mobile communication program on reproductive and child health outcomes: a randomised controlled trial in India. BMJ Glob Health.

[57] Staiano AE, Newton RL, Beyl RA (2022). mHealth intervention for motor skills: a randomized controlled trial. Pediatrics.

[58] Acar Z, Şahin N (2024). Development of a mobile application -based breastfeeding program and evaluation of its effectiveness. J Pediatr Nurs.

[59] Hmone MP, Li M, Agho KE, Alam NA, Chad N, Dibley MJ (2023). Tailored text messages to improve breastfeeding practices in Yangon, Myanmar: the M528 individually randomized controlled trial. Am J Clin Nutr.

[60] Alexandrou C, Henriksson H, Henström M (2023). Effectiveness of a smartphone app (MINISTOP 2.0) integrated in primary child health care to promote healthy diet and physical activity behaviors and prevent obesity in preschool-aged children: randomized controlled trial. Int J Behav Nutr Phys Act.

[61] Wu Q, Wang X, Zhang J, Zhang Y, van Velthoven MH (2023). The effectiveness of a WeChat-based self-assessment with a tailored feedback report on improving complementary feeding and movement behaviour of children aged 6-20 months in rural China: a cluster randomized controlled trial. Lancet Reg Health West Pac.

[62] Vila-Candel R, Mena-Tudela D, Franco-Antonio C, Quesada JA, Soriano-Vidal FJ (2024). Effects of a mobile application on breastfeeding maintenance in the first 6 months after birth: randomised controlled trial (COMLACT study). Midwifery.

[63] Moon RY, Hauck FR, Colson ER (2017). The effect of nursing quality improvement and mobile health interventions on infant sleep practices: a randomized clinical trial. JAMA.

[64] Davis KE, Klingenberg A, Massey-Stokes M (2023). The baby bites text messaging project with randomized controlled trial: texting to improve infant feeding practices. Mhealth.

[65] Saucedo Baza A, Mignacca C, Delgado PE (2023). A technological approach to improved breastfeeding rates and self-efficacy: a randomized controlled pilot study. J Hum Lact.

[66] Bakırcı-Taylor AL, Reed DB, McCool B, Dawson JA (2019). mHealth improved fruit and vegetable accessibility and intake in young children. J Nutr Educ Behav.

[67] Palacios C, Campos M, Gibby C, Meléndez M, Lee JE, Banna J (2018). Effect of a multi-site trial using short message service (SMS) on infant feeding practices and weight gain in low-income minorities. J Am Coll Nutr.

[68] Mindell JA, Du Mond CE, Sadeh A, Telofski LS, Kulkarni N, Gunn E (2011). Efficacy of an internet-based intervention for infant and toddler sleep disturbances. Sleep.

[69] Lewkowitz AK, López JD, Carter EB (2020). Impact of a novel smartphone application on low-income, first-time mothers’ breastfeeding rates: a randomized controlled trial. Am J Obstet Gynecol MFM.

[70] de Mello Sa SR, Wang Z, Sapkalova V (2025). A smartphone-based application to improve breastfeeding duration and self-efficacy: a randomized controlled clinical trial. Women Health.

[71] Sandborg J, Downing KL, Orellana L (2025). Six-month intervention effect of a digital movement behavior intervention on parent- and child intermediary outcomes-results from the Let’s Grow randomized controlled trial. Int J Behav Nutr Phys Act.

[72] Li Y, Xiao Q, Chen M (2024). Improving parental health literacy in primary caregivers of 0- to 3-year-old children through a WeChat official account: cluster randomized controlled trial. JMIR Public Health Surveill.

[73] Jongpaiboonpatana P, Jirakran K, Trairatvorakul P, Chonchaiya W (2025). Increasing parent-child play frequency via exclusively online, video-based, peer-to-peer modeling program: randomized controlled trial. Pediatr Res.

[74] Hunsrisakhun J, Naorungroj S, Tangkuptanon W, Wattanasit P, Pupong K, Pithpornchaiyakul S (2025). Impact of oral health chatbot with and without toothbrushing training on childhood caries. Int Dent J.

[75] Hojati A, Abbasalizad Farhangi M (2025). *MyKid’sNutrition* mobile application: effect on mothers’ nutritional knowledge and nutritional status of preschool-aged children with undernutrition – a randomised controlled trial. BMJNPH.

[76] Henshaw E, Cooper M, Wood T, Krishna S, Lockhart M, Doan S (2024). A randomized controlled trial of the happy, healthy, loved personalized text-message program for new parent couples: impact on breastfeeding self-efficacy and mood. BMC Pregnancy Childbirth.

[77] Gilano G, Dekker A, Fijten R (2025). The Effect of mHealth on exclusive breastfeeding and its associated factors among women in South Ethiopia: a cluster randomized controlled trial. Nutrients.

[78] Cunningham K, Cech S, Gupta AS, Rana PP, Humphries D, Frongillo EA (2026). Text messages to improve young child diets: results from a cluster-randomized controlled trial in Kanchanpur, Nepal. Matern Child Nutr.

[79] Cherie N, Wordofa MA, Debelew GT (2025). The effect of an interactive mobile health intervention to improve community-based essential neonatal care practices among postpartum women in northeast Ethiopia: a cluster randomized controlled trial. Int Health.

[80] Brown AL, Hudson N, Pinfold J (2025). The impact of dose in an mHealth intervention to support parents and carers via healthy beginnings for hunter new England kids program: pragmatic randomized controlled trial. JMIR Form Res.

[81] Phillips SM, Bourke M, Inniss BV, Ahluwalia M, Tucker P (2026). A pilot effectiveness study of a just-in-time micro-randomized controlled trial on the physical activity and sedentary time of young children and their parents: the active family m-health intervention. PLoS ONE.

[82] Gavine A, Marshall J, Buchanan P (2022). Remote provision of breastfeeding support and education: systematic review and meta-analysis. Matern Child Nutr.

[83] Ziebart M, Kammermeier M, Koletzko B, Patro-Golab B (2025). Mobile applications for promoting and supporting breastfeeding: systematic review and meta-analysis. Matern Child Nutr.

[84] Qian J, Wu T, Lv M (2021). The value of mobile health in improving breastfeeding outcomes among perinatal or postpartum women: systematic review and meta-analysis of randomized controlled trials. JMIR Mhealth Uhealth.

[85] Halvorsrud K, Kucharska J, Adlington K (2021). Identifying evidence of effectiveness in the co-creation of research: a systematic review and meta-analysis of the international healthcare literature. J Public Health (Oxf).

[86] Skivington K, Matthews L, Simpson SA (2024). A new framework for developing and evaluating complex interventions: update of medical research council guidance. Int J Nurs Stud.

[87] Henriksson P, Migueles JH, Söderström E, Sandborg J, Maddison R, Löf M (2022). User engagement in relation to effectiveness of a digital lifestyle intervention (the HealthyMoms app) in pregnancy. Sci Rep.

[88] Lippke S, Corbet JM, Lange D, Parschau L, Schwarzer R (2016). Intervention engagement moderates the dose-response relationships in a dietary intervention. Dose Response.

[89] Elkes J, Cro S, Batchelor R (2024). User engagement in clinical trials of digital mental health interventions: a systematic review. BMC Med Res Methodol.

