# Supplementary material for: Digital Interventions Targeting Parents to Improve Early Childhood Movement, Nutrition, and Sleep Behaviors: Systematic Review
Source: J Med Internet Res. 2026 Jun 26;28:e85525. doi: 10.2196/85525 (PMC13309061; doi:10.2196/85525)
Supplement: Multimedia Appendix 1 [file jmir-v28-e85525-s001.pdf]

Multimedia Appendix 1. Full Search Strategy

This appendix provides the complete search strategies used for all databases included in the review. The keyword-based search strategy was run identically across all EBSCO databases (*Academic Search Complete, CINAHL Complete, Global Health, MEDLINE Complete, PsycINFO,* and *SPORTDiscus*) (**Table 1**). For *Embase* (Elsevier), the same conceptual strategy was applied using equivalent database-specific indexing and syntax (**Table 2**). All searches were conducted in December 2022 and updated in August 2024 and January 2026.

Table 1. EBSCO full search strategy.

#	Query
S1	(MH "Physical Activity") OR (MH "Sports+") OR (MH "Exercise") OR (MH "Recreation") OR (MH "Leisure Activities") OR (MH "Bicycles") OR (MH "Games, Recreational") OR (MH "Team Sports") OR (MH "Sitting") OR (MH "Motor Activity") OR (MH "Life Style, Sedentary"))
S2	TI('physical* activ*' OR 'active play' OR 'physical* play' OR 'active transport' OR 'active games' OR sport* OR 'motor activit*' OR 'locomotor activit*' OR accelerom* OR sedentar* OR sitting OR 'physical* inactiv*' OR exercis* OR 'leisure activit*' OR recreation*) OR AB('physical* activ*' OR 'active play' OR 'physical* play' OR 'active transport' OR 'active games' OR sport* OR 'motor activit*' OR 'locomotor activit*' OR accelerom* OR sedentar* OR sitting OR 'physical* inactiv*' OR exercis* OR 'leisure activit*' OR recreation*)
S3	TI(('tummy time' OR (tummy OR prone OR abdomen OR stomach OR belly OR front) N2 (position*)) OR AB(('tummy time' OR (tummy OR prone OR abdomen OR stomach OR belly OR front) N2 (position*))
S4	(MH "Screen Time")
S5	TI('screen time' or screen-time or 'screen based' or screen-based or 'screen use' OR 'electronic media' OR television OR TV OR 'electronic game*' OR e-game* OR 'e game*') OR AB('screen time' or screen-time or 'screen based' or screen-based or 'screen use' OR 'electronic media' OR television OR TV OR 'electronic game*' OR e-game* OR 'e game*')
S6	TI(restraint* or restrained or 'restricted movement' or stroller* or 'high chair*' or 'play pen*' or playpen* or 'baby carrier*' or 'car seat*') OR AB(restraint* or restrained or 'restricted movement' or stroller* or 'high chair*' or 'play pen*' or playpen* or 'baby carrier*' or 'car seat*')
S7	(MH "sleep")
S8	TI(sleep* OR nap OR napping OR naps) OR AB(sleep* OR nap OR napping OR naps)
S9	(MH “Child Nutrition Sciences”) OR (MH “nutritional status”) OR (MH “Diet, Food, and Nutrition”) OR (MH “eating”)
S10	TI(Nutrition* or diet* or (health* N2 eat*) or feed or feeding or ((fruit* or vegetable*) N1 (intake* or consumption or consum*)) or 'family food*' or 'Family meal*' or 'Food habit*' or 'eating habit*' or 'diet* modification' or 'diet* habit*' or 'beverage*' or 'drink*' or 'energy' or 'kilojoule*' or 'calorie*') OR AB(Nutrition* or diet* or (health* N2 eat*) or feed or feeding or ((fruit* or vegetable*) N1 (intake* or consumption or consum*)) or 'family food*' or 'Family meal*' or 'Food habit*' or 'eating habit*' or 'diet* modification' or 'diet* habit*' or 'beverage*' or 'drink*' or 'energy' or 'kilojoule*' or 'calorie*')
S11	(MH “Breast feeding”) OR (MH “bottle feeding”) OR (MH “feeding behavior”) OR (MH “infant formula”) OR (MH “feeding methods”)
S12	TI(breastfe* or (breast* N1 (duration or exclusiv* or optimal*)) or 'formula fe*' or 'formula milk*' OR 'bottle fe*' OR 'infant formula' OR 'feeding behavio#r' OR 'food intake' OR 'artificial milk' OR 'feeding method*') OR AB(breastfe* or (breast* N1 (duration or exclusiv* or optimal*)) OR 'formula fe*' OR 'formula milk*' OR 'bottle fe*' OR 'infant formula' OR 'feeding behavio#r' OR 'food intake' OR 'artificial milk' OR 'feeding method*')
S13	(MH “weaning”)

S14	TI (Weaning or ((infant or baby or first) N1 food*) or (introduc* N2 (solid* or semi-solid* or soft)) or ((solid* or semi-solid* or soft) N3 (food* or feed* or diet*)) or ((compl#mentary or supplementary) and (food* or feed* or nutrition*))) OR AB(Weaning or ((infant or baby or first) N1 food*) or (introduc* N2 (solid* or semi-solid* or soft)) or ((solid* or semi-solid* or soft) N3 (food* or feed* or diet*)) or ((compl#mentary or supplementary) and (food* or feed* or nutrition*)))
S15	S1 OR S2 OR S3 OR S4 OR S5 OR S6 OR S7 OR S8 OR S9 OR S10 OR S11 OR S12 OR S13 OR S14
S16	(MH "Infant") OR (MH "Child+") OR (MH "Child, Preschool")
S17	TI(infan* or baby or babies or toddler* or child* or 'early years' or preschool* or 'pre school*' or kindergarten* or kid or kids or p#ediatric) OR AB(infan* or baby or babies or toddler* or child* or 'early years' or preschool* or 'pre school*' or kindergarten* or kid or kids or p#ediatric)
S18	S16 OR S17
S19	(MH “Behavior Therapy") OR (MH "Early Intervention, Educational") OR (MH “Health Education") OR (MH “Health Promotion") OR (MH “Counseling") OR (MH “Family Therapy") OR (MH “Preventive Medicine") OR (MH “Primary Prevention") OR (MH “social support”) or (MH “cognitive behaviour therapy) OR (MH “health behavior”) OR (MH “behavioral medicine”) OR (MH “psychosocial intervention”) OR (MH “Preventive Health Services” OR (MH “Parenting”)
S20	TI(intervention* or program* or ‘Health behavio* change’ or ‘Behavio* therapy’ or ‘behavio* modification’ OR ‘Health education’ OR ‘health behavio*’ or ‘Health promot*’ or ‘preventive health' OR prevention OR counsel* OR parenting OR 'social support') OR AB(intervention* or program* or ‘Health behavio* change’ or ‘Behavio* therapy’ or ‘behavio* modification’ OR ‘Health education’ OR ‘health behavio*’ or ‘Health promot*’ or ‘preventive health' OR prevention OR counsel* OR parenting OR 'social support')
S21	S19 OR S20
S22	(MH "Internet-Based Intervention") OR (MH "digital technology") OR (MH "telemedicine") OR (MH "Remote Consultation") OR (MH "telenursing")
S23	TI(mhealth OR 'm health' OR ehealth OR 'e health' OR 'e-health' OR 'mobile health' OR (digital N3 health) OR telehealth OR tele-health OR telemedicine OR tele-medicine OR telenursing OR tele-nursing OR 'telephone counselling' OR 'remote health' OR 'virtual health' OR 'online health' OR 'digital technolog*' OR 'text messag*' or SMS or texting or 'instant messag*' or chatbot or 'chat bot' or 'conversational agent*' or (online N3 chat) OR 'dialog* system' OR 'virtual agent' OR 'social media' OR Facebook OR Instagram OR TikTok OR tik-tok OR 'tik tok' OR pinterest OR snapchat OR twitter OR whatsapp OR wechat OR reddit OR ((online OR internet OR discussion) N3 forum*) OR ((mobile OR cell OR cellular OR smart) N3 (phone* OR device*)) OR smartphone* OR iphon* OR android OR (windows N3 (phone OR device)) OR ipad* OR app OR apps OR application* OR webapp OR 'web-app' OR 'web-based' OR 'internet-based' OR 'online platform' OR 'wearable technology' OR 'wearable technologies' OR 'fitness tracker*' OR fitbit* OR 'activity tracker') OR AB(mhealth OR 'm health' OR ehealth OR 'e health' OR 'e-health' OR 'mobile health' OR (digital N3 health) OR telehealth OR tele-health OR telemedicine OR tele-medicine OR telenursing OR tele-nursing OR 'telephone counselling' OR 'remote health' OR 'virtual health' OR 'online health' OR 'digital technolog*' OR 'text messag*' or SMS or texting or 'instant messag*' or chatbot or 'chat bot' or 'conversational agent*' or (online N3 chat) OR 'dialog* system' OR 'virtual agent' OR

	'social media' OR Facebook OR Instagram OR TikTok OR tik-tok OR 'tik tok' OR pinterest OR snapchat OR twitter OR whatsapp OR wechat OR reddit OR ((online OR internet OR discussion) N3 forum*) OR ((mobile OR cell OR cellular OR smart) N3 (phone* OR device*)) OR smartphone* OR iphon* OR android OR (windows N3 (phone OR device)) OR ipad* OR app OR apps OR application* OR webapp OR 'web-app' OR 'web-based' OR 'internet-based' OR 'online platform' OR 'wearable technology' OR 'wearable technologies' OR 'fitness tracker*' OR fitbit* OR 'activity tracker')
S24	S22 OR S23
S25	(MH “randomized controlled trials") OR (MH “clinical study”) OR (MH “evaluation study”) OR (MH “program evaluation”) OR (MH “Health Services research”) OR (MH “comparative study") OR (MH “Cross-Over Studies") OR (MH “pilot projects”) OR (MH “feasibility studies”)
S26	TI('clinical trial' or 'randomi#ed’ or rct or 'comparative stud*' or 'comparison stud*' or 'cross over stud*' or 'crossover stud*' or 'evaluation stud*' OR ((evaluated OR evaluate OR evaluating OR assessed OR assess) AND (compare OR compared OR comparing OR comparison)) or pre-post OR 'pre post' OR ‘pretest-posttest’ OR (pre N3 post) OR quasi OR 'case-control' OR 'case control' OR 'non random*' OR 'non-random*' OR pilot OR feasibility) OR AB('clinical trial' or 'randomi#ed’ or rct or 'comparative stud*' or 'comparison stud*' or 'cross over stud*' or 'crossover stud*' or 'evaluation stud*' OR ((evaluated OR evaluate OR evaluating OR assessed OR assess) AND (compare OR compared OR comparing OR comparison)) or pre-post OR 'pre post' OR ‘pretest-posttest’ OR (pre N3 post) OR quasi OR 'case-control' OR 'case control' OR 'non random*' OR 'non-random*' OR pilot OR feasibility)
S27	S25 OR S26
S28	S15 AND S18 AND S21 AND S24 AND S27

Table 2. EMBASE (Elsevier) full search strategy.

#	Query
S1	'physical activity'/exp OR 'sport'/exp OR 'exercise'/exp OR 'recreation'/exp OR 'leisure'/exp OR 'bicycle'/exp OR 'recreational game'/exp OR 'team sport'/exp OR 'sitting'/exp OR 'motor activity'/exp or 'sedentary lifestyle'/exp
S2	'physical* activ*':ab,ti OR 'active play':ab,ti OR 'physical* play':ab,ti OR 'active transport':ab,ti OR 'active games':ab,ti OR sport*:ab,ti OR 'motor activit*':ab,ti OR 'locomotor activit*':ab,ti OR accelerom*:ab,ti OR sedentar*:ab,ti OR sitting:ab,ti OR 'physical* inactiv*':ab,ti OR exercis*:ab,ti OR 'leisure activit*':ab,ti OR recreation*:ab,ti
S3	'tummy time' OR ((tummy OR prone OR abdomen OR stomach OR belly OR front) NEAR/2 position*)
S4	'screen time'/exp
S5	'screen time':ab,ti OR 'screen based':ab,ti OR 'screen use':ab,ti OR 'electronic media':ab,ti OR television:ab,ti OR tv:ab,ti OR 'electronic game*':ab,ti OR 'e-game*':ab,ti
S6	restraint*:ab,ti OR restrained:ab,ti OR 'restricted movement':ab,ti OR stroller*:ab,ti OR 'high chair*':ab,ti OR 'play pen*':ab,ti OR playpen*:ab,ti OR 'baby carrier*':ab,ti OR 'car seat*':ab,ti
S7	'sleep'/exp
S8	sleep*:ab,ti OR nap:ab,ti OR napping:ab,ti OR naps:ab,ti
S9	'child nutrition'/exp OR 'nutritional status'/exp OR 'nutrition'/exp OR 'eating'/exp
S10	nutrition*:ab,ti OR diet*:ab,ti OR ((health* NEAR/2 eat*):ab,ti) OR feed:ab,ti OR feeding:ab,ti OR ((fruit* OR vegetable*) NEAR/1 (intake* OR consumption OR consum*)):ab,ti OR 'family food*':ab,ti OR 'family meal*':ab,ti or 'food habit*':ab,ti OR 'eating habit*':ab,ti OR 'diet* modification':ab,ti OR 'diet* habit*':ab,ti OR 'beverage*':ab,ti OR 'drink*':ab,ti or 'energy':ab,ti OR 'kilojoule*':ab,ti OR 'calorie*':ab,ti
S11	'breastfeeding'/exp OR 'bottle feeding'/exp OR 'feeding behavior'/exp OR 'artificial milk'/exp OR 'food intake'/exp
S12	breastfe*:ab,ti OR ((breast* NEAR/1 (duration or exclusive* OR optimal*)):ab,ti) OR 'formula fe*':ab,ti OR 'formula milk*':ab,ti OR 'bottle fe*':ab,ti OR 'infant formula':ab,ti OR 'feeding behavio?r':ab,ti OR 'food intake':ab,ti OR 'artificial milk':ab,ti OR 'feeding method*':ab,ti
S13	'weaning'/exp
S14	weaning:ab,ti OR (((infant OR baby OR first) NEAR/1 food*):ab,ti) OR ((introduc* NEAR/2 ('solid*or semi-solid*' OR soft)):ab,ti) OR (((solid* OR 'semi solid*' OR soft) NEAR/3 (food* OR feed* OR diet*)):ab,ti) OR ((compl?mentary:ab,ti OR supplementary:ab,ti) AND (food*:ab,ti OR feed*:ab,ti OR nutrition*:ab,ti))
S15	1 OR 2 OR 3 OR 4 OR 5 OR 6 OR 7 OR 8 OR 9 OR 10 OR 11 OR 12 OR 13 OR 14
S16	'infant'/exp OR 'child'/exp OR 'preschool child'/exp OR 'toddler'/exp OR 'baby'/exp
S17	infan*:ab,ti OR baby:ab,ti OR babies:ab,ti OR toddler*:ab,ti OR child*:ab,ti OR 'early years':ab,ti OR preschool*:ab,ti OR 'pre school*':ab,ti OR kindergarten*:ab,ti OR kid:ab,ti OR kids:ab,ti OR p?ediatric:ab,ti
S18	16 OR 17
S19	'behavior therapy'/exp OR 'early childhood intervention'/exp OR 'health education'/exp OR 'health promotion'/exp OR 'counseling'/exp OR 'family therapy'/exp OR 'preventive medicine'/exp OR 'social support'/exp OR 'cognitive

	behavioral therapy'/exp OR 'health behavior'/exp OR 'behavioral medicine'/exp OR 'psychosocial intervention'/exp OR 'preventive health service'/exp OR 'child parent relation'/exp
S20	intervention*:ab,ti OR program*:ab,ti OR 'health behavio* change':ab,ti OR 'behavio* modification':ab,ti OR 'health education':ab,ti OR 'health behavio*':ab,ti OR 'health promot*':ab,ti OR 'preventive health':ab,ti OR prevention:ab,ti OR counsel*:ab,ti OR parenting:ab,ti OR 'social support':ab,ti
S21	19 OR 20
S22	'web-based intervention'/exp OR 'digital technology'/exp OR 'telemedicine'/exp OR 'teleconsultation'/exp OR 'telenursing'/exp
S23	mhealth OR 'm health' OR 'e health' OR 'e-health' OR 'mobile health' OR (digital NEAR/3 health) OR telehealth OR 'tele health' OR telemedicine OR 'tele medicine' OR telenursing OR 'tele nursing' OR 'telephone counselling' OR 'remote health' OR 'virtual health' OR 'online health' OR 'remote consultation' OR 'digital technolog*' OR 'text messag*' OR sms OR texting OR 'instant messag*' OR chatbot OR 'chat bot' OR 'conversational agent*' OR (online NEAR/3 chat) OR 'dialog* system' OR 'virtual agent' OR 'social media' OR facebook OR Instagram OR tiktok OR 'tik tok' OR pinterest OR snapchat OR twitter OR whatsapp OR wechat OR reddit OR ((online OR internet OR discussion) NEAR/3 foum*) OR ((mobile OR cell OR cellular OR smart) NEAR/3 (phone* OR device*)) OR smartphone* OR iphon* OR android OR (windows NEAR/3 (phone OR device)) OR ipad OR app OR apps OR application* OR webapp OR 'web-app' OR 'web-based' OR 'internet-based' OR 'online platform' OR 'wearable technology' OR 'wearable technologies' OR 'fitness tracker*' OR fitbit OR 'activity tracker'
S24	22 OR 23
S25	'randomized controlled trial'/exp OR 'clinical study'/exp OR 'evaluation study'/exp OR 'comparative study'/exp OR 'program evaluation'/exp OR 'health services research'/exp OR 'crossover procedure'/exp OR 'pilot study'/exp or 'feasibility study'/exp
S26	'clinical trial' OR 'randomi?ed' OR rct OR 'comparative stud*' OR 'comparison stud*' OR 'cross over stud*' OR 'crossover stud*' OR 'evaluation stud*' OR ((evaluated OR evaluate OR evaluating OR assessed OR assess) AND (compare OR compared OR comparing OR comparison)) OR 'pre post' OR 'pretest-posttest' OR (pre NEAR/3 post) OR quasi OR 'case-control' OR 'case control' OR 'non random*' OR 'non-random*' OR pilot OR feasibility
S27	25 OR 26
S28	15 AND 18 AND 21 AND 24 AND 27
S29	15 AND 18 AND 21 AND 24 AND 27 AND ([article]/lim OR [article in press]lim) AND [English]/lim AND [humans]/lim AND [01-12-2022/sd NOT [05-07-2024]/sd
