# Supplementary material for: Digital Interventions Targeting Parents to Improve Early Childhood Movement, Nutrition, and Sleep Behaviors: Systematic Review
Source: J Med Internet Res. 2026 Jun 26;28:e85525. doi: 10.2196/85525 (PMC13309061; doi:10.2196/85525)
Supplement: Multimedia Appendix 2 [file jmir-v28-e85525-s002.pdf]

**Multimedia Appendix 2:** Detailed characteristics of included studies

1. **Table S2.** Intervention details for autonomously delivered digital interventions focusing on pregnancy to infancy (0–11 months) included in this systematic review (n=24).
2. **Table S3**. Intervention details for autonomously delivered digital interventions focusing on toddlers (12–35 months) included in this systematic review (n=6).
3. **Table S4**. Intervention details for autonomously delivered digital interventions focusing on preschool-aged children (36–59 months) included in this systematic review (n=8).
4. **Table S5.** Detailed effectiveness outcomes for digital interventions focusing on pregnancy to infancy (0–11 months) (n=24).
5. **Table S6.** Detailed effectiveness outcomes for digital interventions focusing on toddlers (12–35 months) (n=6).
6. **Table S7**. Detailed effectiveness outcomes for digital interventions focusing on preschool-aged children (36–59 months) (n=8).
7. **Tables S8.** Co-design, process evaluation and engagement outcomes for interventions focusing on pregnancy to infancy (0–11 months) (n=24).
8. **Table S9.** Co-design, process evaluation and engagement outcomes for interventions focusing on toddlers (12-35 months) (n=6).
9. **Table S10.** Co-design, process evaluation and engagement outcomes for interventions focusing on preschoolers (36–59 months) (n=8).

**Table S2.** for autonomously delivered digital interventions focusing on pregnancy to infancy[1] (0–11 months) included in this systematic review (n=24).

| Author (date), reference | Treatment groups | Description of Control | Description of Intervention |
|---|---|---|---|
| **BREASTFEEDING** | | | |
| Ahmed et al. (2016) [1] | 2 | Standard care including breastfeeding support and education before discharge, one phone call within the first week after hospital discharge, list of community breastfeeding resources and access to lactation specialist post-discharge. | Standard care plus access to a computer application for the interactive breastfeeding monitoring system before hospital discharge. Mothers were asked to enter their breastfeeding data, newborn's wet and dirty diapers, and any problems for at least 30 days. The system automatically sent feedback via notifications with tailored interventions if the mother entered data that indicated breastfeeding problems. Educational resources were available through the system (i.e., feeding cues, milk supply management, jaundice management, correct latching, pumping, and returning to work/school). |
| Unger et al. (2018) [2] | 3 | Routine clinic-based counselling and care. | Women in both intervention groups were registered into the Mobile WACh SMS delivery platform and indicated their preferences for message delivery including their name, language (English or Kiswahili), and day of the week and time for delivery. Two different intervention groups: 1) the one-way group received weekly 'push' educational and motivational SMS; 2) the two-way group received the same weekly SMS; however, each SMS contained a question related to the content and the study nurse was available to answer SMS daily on weekdays. |
| Wu et al. (2020) [3] | 2 | Asked to follow the WeChat account called Huzhu County Maternal and Child Health Family Planning Service Centre on their smartphone | The feeding messages were designed for breastfeeding promotion education and provided key breastfeeding knowledge and relevant infant feeding advice, breastfeeding problems encountered for both mother and child, and preparation for both breastfeeding and complementary feeding. All messages were developed based on the WHO breastfeeding recommendations, guidelines, or published literature; messages were published before recruitment. Women could read all messages whenever they wanted. An additional 3 sets of tailored messages were sent via WeChat in late pregnancy (>week 37), the first month postpartum, and 4 months postpartum. Women could participate in the feeding knowledge competition component to test their breastfeeding knowledge and could enter their children's weight and height in the baby growth chart component whenever they want to monitor their children's growth and ask breastfeeding related questions on the online forum component. |
| Lewkowitz et al. (2020) [4] | 2 | A rudimentary novel control app containing only digital versions of conventional breastfeeding support handouts provided at routine third trimester prenatal care visits. The handouts included written education on breastfeeding benefits and availability of in-person breastfeeding resources during their delivery hospitalization and in the postpartum period. | The Breastfeeding Friend (BFF) app including interactive advice on overcoming common breastfeeding challenges, education content on breastfeeding benefits, normal infant behavior and maternal postpartum physiology; and diet and exercise recommendations, strategies to optimize breastfeeding and pumping at work or school, hyperlinks to on-demand videos (e.g., tips for successful latch, troubleshooting latch difficulties, common breastfeeding positions), and hyperlinks to local, national, and international breastfeeding resources. |
| Scott et al. (2021) [5] | 4 | Usual care and attended the breastfeeding component of the hospital-based couple's and antenatal class | Three interventions: 1) a face-to-face father-focused antenatal breastfeeding class (FFABC) facilitated by a male peer; 2) Milk Man, a breastfeeding smartphone app designed specifically for fathers which used gamification, social connectivity in the form |

| | | | |
|---|---|---|---|
| | | | of a conversation forum, and twice-weekly push notifications linking to polls and conversation starters to engage fathers with breastfeeding information contained within an information library which also contained information on all the topics introduced in the FFABC as well as additional breastfeeding and parenting information and links to external websites; and 3) a combination of both interventions. |
| Saucedo Baza et al. (2022) [6] | 2 | Usual care (nursing assistance, physician assistance, lactation consultation, handouts) | The smartphone application (Breastfeeding at AU) plus the usual care offered to women that are breastfeeding (nursing assistance, physician assistance, lactation consultation, handouts). The app contained educational content (information, videos, infographics) which included the following: benefits of breastfeeding. a timeline of infant needs during breastfeeding, maternal products necessary for, or beneficial to, the breastfeeding process, suggested strategies to assist working participants during breastfeeding, answers to commonly asked questions regarding breastfeeding, and information about resources available at the institution for breastfeeding patients. |
| Doan et al. (2022) [7] | 2 | The control group version of the mobile application included information and messages on maternal and child health care in general | A smartphone application with text notifications with behavior change messages (3 times/week during pregnancy; 2 times/week after delivery) and suggestion for further resources in the application's library. During pregnancy, 3 messages were auto-generated weekly, and 12 key messages were repeated after 4 weeks. The set of 12 key messages targeted at both mothers and family members were developed based on target behaviors, key determinants, and improving breastfeeding self-efficacy theories, and were delivered during the prenatal period. Messages emphasizing the importance of breastfeeding exclusively were reinforced at 1 and 4 months. Some of the messages sent after birth addressed common breastfeeding problems, including sore nipples, breast engorgement, low milk supply, and mastitis. |
| LeFevre et al. (2022) [8] | 2 | No messages | Kilkari is comprised of 90 min of reproductive, maternal, neonatal and child health content sent via 72 once weekly voice calls: 24 during pregnancy, 24 within the first 6 months postpartum and 24 from 7 to 12 months postpartum. |
| Acar and Sahin (2023) [9] | 2 | Routine postpartum care | For the first use of the mobile application-based breastfeeding program (MABBP), the app included three pages with information about the importance of breastmilk, WHO breastfeeding recommendations, and instructions on the use of the application. After these informative pages, there was a screen with the mother's name, baby's name, baby's gender, and the baby's weight. The main screen of the application consisted of six screens. These screens were: “Breastfeeding” (breast milk characteristics, the importance of breastfeeding, breastfeeding technique, etc.), “Milking” (milking by hand, milking with a pump, milk storage conditions, etc.), “Unexpected Situations” (breastfeeding problems and solution suggestions), “Frequently Asked Questions”, “Breastfeeding Entry,” and “Breastfeeding Diary” tabs. The “Breastfeeding Entry” tab allowed mothers to track their breastfeeding status and record the number and length of each breastfeeding period, as well as their milking status, the baby's height, and weight measurements each month, and any breastfeeding-related issues they encountered. Additionally, weekly notifications were sent via the app to increase mothers' breastfeeding motivation. |
| Hmone et al. (2023) [10] | 2 | The control group received maternal and child health care messages, excluding breastfeeding-related information, once a week. | Over ~38 week, from recruitment to 6 months postpartum, the intervention group received breastfeeding promotional text messages 3 times/wk. The texts were from a trusted source, short, locally acceptable, memorable, and with actionable content. |

| | | | |
|---|---|---|---|
| Vila-Candel et al. (2024) [11] | 2 | Standard care including individual counselling about the benefits of maintaining breastfeeding for the first 6 months of the baby's life and introducing complementary foods. The mother was required to attend at least six scheduled visits, including two with the midwife and four with the primary care pediatrician before the baby reached 6 months of age | A free mobile application (Lactapp, automated breastfeeding consultation system, based on self-administered questionnaire and official health recommendations) which provides personalized support to women about breastfeeding. Following registration in the app (during the third trimesters), users can access up-to-date information supported by expert lactation professionals, allowing them to make informed decisions and resolve doubts before or after birth. The application allows for breastfeeding tracking by recording e.g., daily feedings, difficulties, and mood, and provides a live chat to enable discussions of breastfeeding concerns. Timely reminders (e.g., breastfeeding positions, feeding frequency, and the significance of exclusive breastfeeding) is sent based on the user's due date. |
| Henshaw et al. (2024) [12] | 2 | Control group received usual care followed by 6 weeks of attentional control text messages about infant development | Couples completed a brief educational tablet program together, followed by 6 weeks of tailored text messages. Message content was mapped to the content in the tablet program, providing reminders, resources and encouragement. Text messages matched mothers' responses to questions in the tablet module. Partners received corresponding text messages to provide support and encouragement to mother. |
| De Mello et al. (2025) [13] | 2 | Standard care only: nursing assistance, physician assistance, lactation consultation, and patient breastfeeding handouts (no app). | Standard care + “Breastfeeding at AU” smartphone app (Apple iPhone/iPad). App provided breastfeeding educational materials and medically accurate info in simple language, including strategies for working mothers, timelines for infant needs, institution-specific resources, and FAQs with answers. App provided from 32–36 weeks gestation (third trimester) onward. |
| Brown et al. (2025) [14] | | No control group | Intervention SMS to increase duration of breastfeeding.<br>Low-dose text message group received up to 2 messages per week in the first month, and approximately 1 message per week from child age 2 months - 2 years<br>High dose text message group received 3-4 messages per week in the first month, 2 messages per week in the second month, and 5 messages per week from child age 3 months - 2 years |
| Cherie et al. (2025) [15] | | Standard healthcare | Intervention SMS to improve neonatal care practices. Intervention group received one SMS every 2 weeks (8 messages in total) in addition to standard healthcare |
| Gilano et al. (2025) [16] | | Standard antenatal, delivery, postnatal and vaccination services as per usual care | Intervention group received 1 health information SMS every 2 weeks for 9 months. Messages focused on antenatal care, postpartum family planning, vaccination, breastfeeding/nutrition tips, tips for maintaining exclusive breastfeeding, danger signs, partner/community engagement |
| **BREASTFEEDING AND FEEDING PRACTICES** | | | |
| Palacios et al. (2018) [17] | 2 | Control SMS related to general infant’s health issues related to sleeping, bathing, teething, traveling in a car, medications, handling baby, and smoking, information related to immunization, and care of common illnesses. | Intervention SMS focused on reinforcing WIC messages on breastfeeding, preventing overfeeding, delaying introduction of solid foods, and delaying and reducing baby juice consumption. A total of 18 messages (1 per week for 4 months) were sent. |
| Davis et al. (2023) [18] | 2 | The control group received usual care—handouts from the American Academy of Paediatrics about safety and feeding. They also received safety-related text messages twice a week for the first 4 weeks, once a week from 5 to 8 weeks, and every 2 weeks for the remainder of the | The intervention group received text messages with feeding advice four times per week for the first 4 weeks of study enrolment, decreasing to twice a week by 5 weeks, and once a week from 8 weeks to 12 months of age. Both groups received the clinic's handouts including feeding and general infant safety advice. Parents who were breastfeeding received messages promoting and supporting breastfeeding. Formula-feeding parents |

| | | | |
|---|---|---|---|
| | | study (up to 12 months of life). Both groups received the clinic's handouts including feeding and general infant safety advice | received messages intended to provide cues to action regarding appropriate feeding practices and timing and to promote feeding self-efficacy. At about 6 months of age, parents received a text to enroll in the introduction to solid foods group. Some messages contained links to a web page where parents could read content with more information on feeding advice. The web page also contained links to external web pages with additional information or videos related to feeding and infant nutrition care. |
| Li et al. (2024) [19] | 2 | Control CHCs received printed educational materials similar to the intervention content. All caregivers subscribed to the "Scientific Parenting" WeChat Official Account (WOA) for survey/data collection, but control CHCs could access the survey module only (no intervention module). Both groups could access routine child health services as usual during follow-up. | Intervention CHCs received the "Scientific Parenting" WeChat Official Account (WOA) program: purpose-designed videos constructed in line with the WHO/Europe parental health literacy model (12 subdimensions). Fifteen topics across health care, disease prevention and health promotion, designed to build caregivers' capacity to access, understand, appraise and apply child health information. Over the 9-month intervention, participants could self-navigate and video views were automatically recorded. Supplementary links to trusted web-based reading materials (e-books, websites, other WOAs) were provided, closely aligned with video topics and also covering areas such as early education and healthy home environment. |
| **COMBINED** | | | |
| Wen et al. (2020) [20] | 3 | Standard care from child and family health nurses in the local health districts. Home safety promotion materials and a newsletter on "Kid's Safety" were sent at the third trimester and at 3, 6, and 9 months of child age. | Intervention group 1: intervention booklet plus six 30-60 min long nurse-led telephone support: Intervention group 2: intervention booklet plus a set of SMS messages sent out twice a week for 4 weeks included key messages from the booklet |
| Wu et al. (2023) [3] | 2 | The movement behavior group acted control for the complementary feeding group and vice versa | Tailored feedback report based on participant questionnaire answers (same questionnaire for both groups), consisting of total scores for all questions, personal feeding or movement behavior problems and corresponding recommendations (also shown in pictures and videos). The feedback reports were different for the two groups: the feeding group received feedback reports based on feeding questions in the questionnaire, and the movement behavior group received feedback reports based on movement behavior questions. |
| **DIET** | | | |
| Roed et al. (2021) [21] | 2 | Not described | Food4toddlers intervention encouraged participants to have core foods available, especially vegetables and fruit, both in their home and on the child's plate. The opposite was encouraged for discretionary foods and beverages. The Food4toddlers website included a website with four elements: modules including 2-4 lessons covering an introduction and seven topics on promoting healthy food and eating environment for the child; recipes; a discussion forum; and highlighted information about food and beverages ("Good to know"). When accessing the website, a video with study information and how important a small weekly increase in vegetable consumption may be for the child's long-term health appeared. The modules included activity elements (e.g., quiz or game), and visual elements supporting that information. In addition, participants received weekly emails containing a link to a new lesson (20 times). The estimated time to complete an average lesson was 10 min. The Food4toddlers website was available on smartphones and other tablets in the form of a mobile app, in addition to computers. |
| Helle et al. (2019) [22] | 2 | Routine care from their local child health clinic with regular consultations at child age 6, 8, 10 and 12 months. | A webpage with a monthly age-appropriate video addressing infant feeding topics together with corresponding cooking films/recipes, were offered to participants in the |

| | | | |
|---|---|---|---|
| | | | intervention group from child age 6 to 12 months. The video clips were of 3–5 minutes duration and focused on feeding-related aspects like appropriate food-types and textures, development of taste-preferences, adequate and varied intake of fruits and vegetables and responsive feeding practices. The cooking-films demonstrated how to make homemade baby- and family food from healthy ingredients in an easy way |
| **SLEEP** | | | |
| Moon et al. (2017) [23] | 4 | 4 intervention combinations: (1) breastfeeding NQI and breastfeeding mHealth; (2) safe sleep NQI and breastfeeding mHealth; (3) breastfeeding NQI and safe sleep mHealth; or (4) safe sleep NQI and safe sleep mHealth.<br><br>The nursing quality improvement (NQI) interventions were designed to ensure that mothers would hear key messages, and that there was appropriate role modelling by hospital personnel. All mothers who delivered at a given hospital were exposed during routine postpartum care to education and role modelling resulting from the assigned NQI intervention, along with any preexisting educational practices performed at the hospital. The NQI used a train-the-trainer model with local nurse champions as coordinators, evidence-based educational materials that provided strategies for addressing barriers to safe sleep and breastfeeding and an emphasis on the importance of role modelling best practices. Each hospital team decided on and implemented NQI initiatives in plan-do-study-act cycles. Following each cycle, hospital staff conducted unannounced audits in which they observed maternal practices and asked mothers about information received from staff.<br><br>Mothers in all groups received basic information about breastfeeding and safe sleep per hospital protocols, including advice to bring the infant into the parental bed for feeding, but to move the infant back into a separate sleep space when the parent was ready for sleep, and to postpone pacifier use for directly breastfed infants until breastfeeding was well established. | The mHealth interventions provided ongoing messaging timed to anticipate likely adherence challenges. Health messages and educational videos were delivered by email or text messages to parents. Videos contained parent testimonials, addressed common questions and barriers to safe sleep (intervention) or breastfeeding (control), and were delivered at times when these issues typically arise. At enrolment while in the hospital, mothers answered questions about current infant feeding status and feeding plans after hospitalization and viewed the first 2 TodaysBaby videos (safe sleep or breastfeeding; each approximately 3 minutes long). Within 24 hours, participants began receiving email or text messages (based on participant preference) with TodaysBaby videos (lasting 60-90 seconds long). Mothers received daily messages and videos for the first 11 days and then every 3 to 4 days for 60 days.<br><br>Mothers in all groups received basic information about breastfeeding and safe sleep per hospital protocols, including advice to bring the infant into the parental bed for feeding, but to move the infant back into a separate sleep space when the parent was ready for sleep, and to postpone pacifier use for directly breastfed infants until breastfeeding was well established. |

[1] 0-2 months of age for newborns and 3-11 months of age for infants.

**Table S3.** Intervention details for autonomously delivered digital interventions focusing on toddlers[1] (12–35 months) included in this systematic review (n=6).

| Author (date), reference | Treatment groups | Control | Intervention |
|---|---|---|---|
| **COMBINED[2]** | | | |
| Alexandrou et al. (2023)[3] [24] | 2 | Standard care offered by the Swedish primary health care system during routine visit at 2.5%3 years. This includes a conversation about healthy foods and eating behaviors and health behaviors in general, as well as a pamphlet with information on healthy lifestyle behaviors. | MINISTOP 2.0 app delivering a program of information over 13 themes (with general information, practical tips and strategies), and registration and weekly feedback for child intakes of fruit and vegetables, sweets, snacks and sugar-sweetened beverages. |
| Sandborg et al. (2025) [25] | 2 | Usual care + eight electronic "Toddler Tips" bulletins emailed every 6 weeks during the intervention period on unrelated topics (e.g., basic child first aid, language development, toilet training). | Let's Grow app (mobile web app) w linked SMS notifications for 12 months. App built around eight learning modules (text + video) covering child sleep, play/physical activity, screen time and general parenting; parents choose module order but can only complete one module at a time. Each module includes behavior change activities supported by SMS prompts (e.g., goal setting, self-monitoring/goal monitoring) and has a minimum completion timeframe (2–5 weeks) before moving to the next module; completed modules remain accessible. App also includes a toolkit (parenting strategies/info) and a community chat forum accessible throughout. |
| Jongpaiboonpatana et al. (2025) [26] | 2 | Waitlist control (no active comparator): parents continued usual activities during the 10-week study period; received assessment prompts via LINE at 6 and 10 weeks and telephone screen-time interview at 10 weeks; intervention videos provided after study completion | PoPPI delivered via LINE (Line Official Account): one 2–3 min video released weekly for 6 weeks; parents could view on-demand and re-watch previous weeks' content. Videos featured a developmental pediatrician and psychologist emphasizing the importance of play and sharing tips to enhance parent–child communication/interaction, plus peer-to-peer modelling using short segments filmed by real parents demonstrating parent–child play activities. Weekly reminder message sent alongside each video via LINE. Intervention underpinned by Bandura's social learning theory (behavior change via observation/modelling) |
| **DIET** | | | |
| Cunningham et al. (2026) [27] | 2 | Suaahara II intervention of 10 key behaviors in first 1000 days (including mother and child diet diversity). Combination of interpersonal communication, community meetings, food demonstrations, community radio program | Suaahara II + text messages |
| Hunsrisakhun et al. (2025) [28] | 2 | Group 1: 10-15 mins of in-person toothbrush training with 21-Day FunDee (modified) chatbot. Chatbot provided 21 daily dialogues which included games, infographics, animations, songs, and general knowledge on oral health care for young children. Also included recommendations for feeding practices and control of sugar consumption. | Group 2: Expanded 21-Day FunDee chatbot. Provided instruction for young children on tooth-brushing techniques. Included interfaces to compare plaque levels. Chatbot was an optional 4-day dialogues which included the significance of deciduous teeth, preparing children for dental visits, necessity of decreasing sugar intake. |
| **SLEEP** | | | |
| Mindell et al. (2011) [29] | 3 | Instructions to follow their child's usual bedtime practices throughout the entire 3-week period. | Internet-based intervention group: An algorithm -based internet intervention, i.e., customized sleep profile (CSP) which collects caregivers' responses on an expanded version of the BISQ and provides parents with individualized information across three |

general domains (normative comparison of their child's sleep to other children of the same age; rating of whether their child is an "excellent, good, or disrupted sleeper"; customized advice on how caregivers can help their child sleep better at night). The provided recommendations are tailored to each child based on the BISQ responses and provided online in text and as graphs, as well as being automatically emailed to each family. Mothers in the internet intervention groups received access to the CSP following completion of all baseline measures on day 8; Internet-based intervention + routine group: In addition to completion of the internet-based intervention these mothers were instructed to institute a nightly 3-step bedtime routine that included a bath (using a provided wash product), a lotion/massage (using a provided moisturizing product), and quiet activities (e.g., cuddling, singing lullaby) with lights out within 30 minutes of the end of the bath.

[1] 12-35 months of age (≥1 to < 3 years).
[2] The combined interventions focused on multiple behaviors e.g., diet, and physical activity/sedentary behavior.
[3] This study focused on both the toddler period and preschool age.

**Table S4.** Intervention details for autonomously delivered digital interventions focusing on preschool-aged children[1] (36–59 months) included in this systematic review (n=8).

| Author (date), reference | Treatment groups | Control | Intervention |
|---|---|---|---|
| **COMBINED [2]** | | | |
| Knowlden et al. (2015) [30] | 2 | Healthy Lifestyles program: A knowledge-based program (active control) including five educational sessions (one session to each child behavior), and a booster session between post-test and follow-up measurement, and educational modalities for each session included a 10-15 min audiovisual presentation, an interactive worksheet, and a discussion board post (same as the EMPOWER program). | The EMPOWER program was designed to reify and improve five SCT constructs in mothers: environment, emotional coping, expectations, self-control and self-efficacy. The program composed of five educational sessions (one session to each child behavior) intended to assist mothers in helping children achieve four behaviors to prevent childhood obesity: 120 minutes of daily, structured and unstructured physical activity; daily consumption of five cups of fruits and vegetables; substitution of sugar-sweetened beverages with sugar-free beverages; and restriction of screen time to no more than 120 min/day. A booster session was delivered between post-test and follow-up measurement. Educational modalities for each session included a 10-15 min audiovisual presentation, an interactive worksheet, and a discussion board post. |
| Delisle-Nyström et al. (2017) [31] | 2 | Pamphlet on healthy eating and physical activity in preschool-aged children based on existing guidelines. | The MINISTOP intervention was delivered through an app and consisted of a program of information and support centered around guidelines for healthy eating and physical activity in preschool-aged children. The intervention included 12 themes (healthy foods in general, breakfast, healthy small meals, physical activity and sedentary behavior, candy and sweets, fruits and vegetables, drinks, eating between meals, fast food, sleep, foods outside the home, and foods at special occasions), with a new theme being introduced biweekly. Each theme consisted of general information, advice, and evidence-based strategies on how to change unhealthy behaviors. Parents were encouraged to register information on their child's intake of fruits, vegetables, candy and sweetened beverages as well as time spent being sedentary once a week. At the end of the week parents received graphic feedback and automated comments based on the submitted information. Parents could also contact a dietitian and/or psychologist to ask questions within the app. |
| Sun et al. (2017) [32] | 2 | Weekly mailing lists of printed health information | Tablet-computer-based intervention consisting of 8 weekly 30-min, interactive, educational modules accessed online via tablet computers, covering the following topics: Introduction to the 5-4-3-2-1-0 program (containing recommendations: 5 servings of fruits and vegetables, 4 servings of water, 3 servings dairy, 2 hours screen time, 1 hour physical activity, 0 sugar drinks); Energy balance - maintain a healthy weight; What to feed my family - energy IN; Grocery shopping; Find fun in physical activity - energy OUT; Less sit, more fit - decrease screen time; Smart parenting; and Maintain a healthy weight for life. |
| Hammersley et al. (2019) [33] | 2 | Fortnightly emails containing links to the Raising Children Network website (an Australian government-funded parenting website). The topics were similar to the intervention (nutrition, physical activity, screen time, and sleep), but no interactive components. Participants were also | The Time2bHealthy program was delivered through website and weekly emails (reminders to log in to the website and participate in the activities), targeted multiple behaviors and consisted of 6 modules (introduction, nutrition [2], physical activity, screen time, sleep). Each module comprised reading material, videos, activities, quizzes, and a goal-setting component. Feedback on participants' goals and advice on how to |

| | | | |
|---|---|---|---|
| | | encouraged to access and contribute to a closed Facebook group to communicate with other members and the dietitian (separate group for the control and intervention)? | improve their goals using the SMART foal framework was provided by a dietitian after each module. Participants were also encouraged to access and contribute to a closed Facebook group to communicate with other members and the dietitian (separate group for the control and intervention). Participants continued to receive emails (including infographics summarizing key points from each module, encouragement to log back into the website to revise the material and review their progress with their goals) fortnightly at the end of the program until the 6-month follow up. |
| **DIET** | | | |
| Bakirci-Taylor et al. (2019) [34] | 2 | 12 text messages about physical activity | Jump2Health mobile intervention included three components: a mobile website, social media (Facebook page where 177 posts with vegetable and fruit content were posted) and 12 text messages about ways to encourage more vegetable and fruit consumption. The Facebook page provided information that was unavailable on the Jump2Health website, but also mentioned and reinforced information and text and promoted linked resources from the website. |
| Hojati et al. (2024) [35] | 2 | Standard care included consultations with pediatric specialist for developmental assessment and personalized health care plan, and nurses to oversee anthropometric measurements and nutrition consultations | MyKid'sNutrtition app contains educational content in four main categories: children's healthy eating, tips and recommendations to overcome children's undernutrition, resolving feeding difficulties in children, and assessing child's growth status. The app also features interactive growth charts for tracking weight-for-age, height-for-age, weight-for-height, and BMI. |
| **PHYSICAL ACTIVITY** | | | |
| Staiano et al. (2022) [36] | 2 | The PLAY app with the Free Play condition: access to lessons and videos that promoted equivalent amount (12 min/day, 5 days/week) of unstructured physical activity that is not dictated or guided by parents. Topics included strategies to make time for and create an environment conducive to the child's free play: setting goals, reinforcing physical activity, being active indoors and outdoors, and reducing sedentary behavior. Parents received automated push notifications 5 times per week to remind them to access the content on the app and ensure their child attained the 12 minutes per day, 5 days per week goal. A point system was built in for the child to select a star for each 12-min period completed, earning up to 5 stars each week. | The PLAY app with two conditions. The Motor skills condition included weekly motor skills instructional lessons, peer modelling videos, and activity breaks to deliver 12 hours of targeted structured motor skills instruction time to their child over a 12-week period (12 min/day, 5 days/week). Parents received automated push notifications 5 times per week to remind them to access the content on the app and ensure their child attained the 12 minutes per day, 5 days per week goal. A point system was built in for the child to select a star for each 12-min period completed, earning up to 5 stars each week. |
| Phillips et al. (2026) [37] | 2 | Control prompts: parents completed the same brief EMA (availability + context questions) as the intervention prompts but received no micro-intervention content (no activity suggestion). | If available at a prompt, parents received an immediate JITAI "micro-intervention" via text: an age-appropriate activity recommendation cue for parent–child co-participation, tailored to context (e.g., indoors/outdoors, who they were with, weather; plus availability checks like with child, child awake, at home, child already active). Seven prompts/day at random times (≥60 min apart) between 7am–7pm for 14 days (98 decision points). Intervention content (selected via branching/randomizers) included activity suggestions, embedded internet videos, and links to publicly available activity resources (73 micro-interventions developed). |

[1] 36-59 months of age (≥3 to < 5 years).
[2] The combined interventions focused on multiple behaviors e.g., diet, and physical activity/sedentary behavior.

**Table S5.** Detailed effectiveness outcomes for digital interventions focusing on pregnancy to infancy[1] (0–11 months) (n=24).

| Author (date), reference | Breastfeeding | Feeding practices | Diet | PA | SB | ST | Sleep |
|---|---|---|---|---|---|---|---|
| **BREASTFEEDING** | | | | | | | |
| Ahmed et al. (2016) [1] | S higher rates of exclusive and partial breastfeeding in the intervention group at 1, 2, and 3 months<br><br>NS for differences in breastfeeding outcomes between groups at discharge | NA | NA | NA | NA | NA | NA |
| Unger et al. (2018) [2] | S higher probability of exclusive breastfeeding in the one-way SMS arm at 10 and 16 weeks, and in the two-way SMS arm at 10, 16 and 24 weeks, compared with controls | NA | NA | NA | NA | NA | NA |
| Wu et al. (2020) [3] | S higher exclusive and predominant breastfeeding in the intervention group at 0-1 month postpartum<br><br>NS for exclusive breastfeeding rate 2-3 months and 4-5 months postpartum | S lower rate of giving dairy products at 0-1 month postpartum in the intervention group.<br><br>NS for giving semisolid or solid foods during the past 24 hours at 0-1 month, 2-3 months and 4-5 months | NA | NA | NA | NA | NA |
| Lewkowitz et al. (2020) [4] | NS for exclusive breastfeeding at postpartum day 2, breastfeeding initiation, rates of nonexclusive and exclusive breastfeeding from 2 days postpartum to 6 | NA | NA | NA | NA | NA | NA |

| | | | | | | | |
|---|---|---|---|---|---|---|---|
| | months postpartum, breastfeeding issues and reasons for formula initiation, etc. | | | | | | |
| Scott et al. (2021) [5] | NS for exclusive breastfeeding and any breastfeeding | NS for introduction of formula, introduction of complementary foods | NA | NA | NA | NA | NA |
| Saucedo Baza et al. (2022) [6] | NS for the rate of exclusive breastfeeding which was nearly twice as high in the intervention group (65%, n=11) as in the control group (32%, n=6) | NA | NA | NA | NA | NA | NA |
| Doan et al. (2022) [7] | S increases for early initiated breastfeeding within 2 hours (adjusted odds ratio [aOR] = 1.50, 95% confidence interval [CI]: 1.01–2.24) and exclusive breastfeeding during hospital stay (aOR= 1.60, 95% CI: 1.03–2.48) for the intervention group.<br><br>NS for exclusive breastfeeding at 1, 4 and 6-months | NA | NA | NA | NA | NA | NA |
| LeFevre et al. (2022) [8] | NS for exclusive breastfeeding | NA | NA | NA | NA | NA | NA |
| Acar and Sahin (2023) [9] | S higher rate of breastfeeding exclusively (test value 4.3 and 5.9 at first and second follow up, respectively) and lower rate of experiencing | NA | NA | NA | NA | NA | NA |

| | | | | | | | |
|---|---|---|---|---|---|---|---|
| | breastfeeding problems (test value -2.5 and -3.9 at the first and second follow up, respectively) vs the control group | | | | | | |
| Hmone et al. (2023) [10] | S higher exclusive breastfeeding prevalence at 6 months (RR: 1.48; 95%CI: 1.35-1.63) and at each monthly visit, and S increased current breastfeeding (RR: 1.17; 95%CI: 1.07-1.26) at 6 months vs the control group. Exclusive breastfeeding and current breastfeeding were progressively higher at each follow up vs the control group. | S reduced bottle feeding (RR: 0.30; 95%CI: 0.17-0.54) at 6 months vs the control group. | NA | NA | NA | NA | NA |
| Vila-Candel et al. (2024) [11] | NS for breastfeeding rate 6-month postpartum compared with standard care (CG = 41.6% vs. IG = 43.6 %; p = 0.826). | NA | NA | NA | NA | NA | NA |
| Henshaw et al. (2024) [12] | NS for exclusive breastfeeding at 6 weeks | NS for breastfeeding self-efficacy at 6 weeks<br><br>S higher breastfeeding self-efficacy at 6 months in intervention group (M=3.69, SE=0.15, 95% CI= 3.39, 3.99) compared to control group (M=3.12, SE=0.18, 95% CI= 2.75, 3.46) | NA | NA | NA | NA | NA |
| De Mello et al. (2025) [13] | NS: Breastfeeding duration (10.1 vs 8.9 | NS: Change in breastfeeding self-efficacy (BSES-SF) from | NA | NA | NA | NA | NA |

| | | | | | | | |
|---|---|---|---|---|---|---|---|
| | months; p=0.320; Kaplan–Meier p=0.241). NS: Exclusive breastfeeding at 6 months (81.3% vs 60.0%; p=0.277). | antenatal to 12 months postpartum not different between groups (p=0.498). | | | | | |
| Brown et al. (2025) [14] | NS for breastfeeding rates | NS for differences in feeding status by dose | NA | NA | NA | NA | NA |
| Cherie et al. (2025) [15] | breastfeeding was n(%): Control: 272 (74.1) Intervention: 289 (76.9) | Only n (%) provided, so significance is unknown Initiated breastfeeding within one hour after birth (control: 273 (74.4); intervention: 321 (85.4)) Baby fed colostrum (control: 294 (80.1), intervention 351 (93.4)) | NA | NA | NA | NA | NA |
| Gilano et al. (2025) [16] | S lower risk of stopping breastfeeding (AHR = 0.40, 95% CI: 0.26-0.62, p<0.001) | S higher odds of breastfeeding in the first hour after birth (AOR = 4.71, 95% CI: 3.10-7.16, p<0.001) | NA | NA | NA | NA | NA |
| **BREASTFEEDING AND FEEDING PRACTICES** | | | | | | | |
| Palacios et al. (2018) [17] | NS for exclusive breastfeeding rates | NS for introduction of other foods and beverages, addition of foods to the bottle, placing infants to sleep with milk bottles, caregiver's method and response to feeding infants and distractions while feeding infants | NA | NA | NA | NA | NA |
| Davis et al. (2023) [18] | NS for breastfeeding persistence | NS for differences in age of introduction of solids in the intervention (statistical analysis not possible due to low counts) vs the control group | NA | NA | NA | NA | NA |
| Li et al. (2024) [19] | S: Higher EBF rate at 6 months in intervention vs control | S: Higher awareness of VD supplementation for infants <6 | NS: iron-fortified staple food | NA | NA | NA | NA |

| | | | | | | | |
|---|---|---|---|---|---|---|---|
| | (38.9% vs 23.44%; RR 1.90, 95% CI 1.07–3.38). NS: BF rate at 12 months. | months (76.7% vs 70.5%; RR 1.39, 95% CI 1.06–1.82). | supplementation at 6 months. | | | | |
| **COMBINED** | | | | | | | |
| Wen et al. (2020) [20] | NS for breastfeeding rates between the groups at both 6 and 12 months | S higher odds of appropriate timing of introducing solid foods (adjusted odds ratio [AOR], 1.68 [95% CI, 1.22-2.32]), cup use (AOR, 1.54 [95% CI, 1.12-2.13]), at 6 months and higher odds of having no bottle at bedtime (AOR, 1.73 [95% CI, 1.23-2.42]) at 12 months. Use of SMS also led to higher odds than the control of having no bottle at bedtime (AOR, 1.29 [95% CI,1.10-1.51]) at 12 months. | NA | NS for daily tummy time frequency at 6 months, and child being active >2h/day at 12 months | NA | S higher odds of having no screen time (AOR, 1.80 [95% CI, 1.28-2.53]) at 12 months. Use of SMS also led to higher odds than the control group of having no screen time (AOR, 1.28 [95% CI, 1.08-1.52]) at 12 months. | NA |
| Wu et al. (2023) [38] | NS for continued breastfeeding at 12–23 months (% of children 12–23 months of age who were fed breast milk during the previous day) | S higher prevalence of minimum diet diversity (OR: 1.62 [95% CI, 1.16–2.28), minimum meal frequency (OR: 1.45 [95% CI, 1.03–2.04) and minimum acceptable diet (OR: 1.51 [95% CI, 1.12–2.05) in the feeding group vs the movement behavior group from baseline to the second follow-up | S increase in monthly intakes of flesh foods (OR: 1.42 [95%CI: 1.03-1.95]), eggs (OR: 1.40 [95%CI: 1.01-1.92]) pulses, nuts and seeds (OR: 1.59 [95%CI: 1.15-2.19]) in the feeding group vs the movement behavior group | S higher improvement in the proportion of children who met physical activity time during the last 24 h at the second follow-up (OR: 2.22 [95% CI, 1.26–2.17]) and outdoor time at the second follow-up (OR: 1.94 [95% CI, 1.49–2.54) in the movement behavior group vs the feeding group | NS for physical restraint | S increase in ST only at the first follow-up (OR: 1.36 [95% CI, 1.02–1.82) in the movement behavior group vs the feeding group | NS |
| **DIET** | | | | | | | |
| Roed et al. (2021) [21] | NA | NA | S larger increase in the frequency of vegetable intake (mean change | NA | NA | NA | NA |

| | | | | | | | |
|---|---|---|---|---|---|---|---|
| | | | 0.46; 95%CI: 0.06-0.86) vs the control group.<br><br>NS for child food intake of fruits, vegetables and discretionary foods between baseline and follow-up 2 | | | | |
| Helle et al. (2019) [22] | NA | S higher food responsiveness and lower emotional over-eating in the intervention group vs control (eating behavior).<br><br>NS - on maternal feeding practices or maternal feeding style | NS for intakes of fruit, vegetables, sweet and salty snacks and soft drinks, for child eating mostly homemade vs commercially prepared dinner, child mealtime habits or frequency of family meals | NA | NA | NA | NA |
| **SLEEP** | | | | | | | |
| Moon et al. (2017) [23] | NA | NA | NA | NA | NA | NA | S higher prevalence of placing their infant's supine, room sharing without bed sharing, no soft bedding use, and any pacifier use among mothers receiving the safe sleep mobile health intervention compared with mothers receiving the control mobile health intervention |

PA, physical activity; SB, sedentary behavior; ST, screen time; NA, not assessed; NS not significant; S, significant; S/NS, some significant and some non-significant results.

**Table S6.** Detailed effectiveness outcomes for digital interventions focusing on toddlers[1] (12–35 months) (n=6).

| Author (date), reference | Breastfeeding | Feeding practices | Diet | PA | SB | ST | Sleep |
|---|---|---|---|---|---|---|---|
| **COMBINED** | | | | | | | |
| Alexandrou et al. (2023) [24] | NA | NA | S lower intakes of sweet and savory treats (-6.97 g/day), sweet drinks (-31.52 g/day), higher intakes of vegetables (2.91 g/day) vs control | NS for MVPA | NA | S less ST (-7.00 min/day) vs control | NA |
| Sandborg et al. (2025) [25] | NA | NA | NA | S: Higher parental knowledge of child movement behaviors at 6 months (total knowledge MD 0.41; 95% CI 0.15–0.67; p=0.002), driven by physical activity knowledge (MD 0.12; 95% CI 0.01–0.22; p=0.028). | NS: No significant effects on other sedentary behavior/screen time-related intermediary outcomes (e.g., self-efficacy/confidence, co-participation, role modelling, rules/routines, screens in bedroom). | NS: Screen time knowledge not different at 6 months (MD 0.03; 95% CI −0.13 to 0.18; p=0.715). | S: Higher sleep knowledge at 6 months (MD 0.27; 95% CI 0.10–0.45; p=0.003). |
| Jongpaiboonpatana et al. (2025) [26] | NA | NA | NA | S: Parent–child play frequency (PPQ) improved vs control (Total score) at 10 weeks (MD 3.48; d=0.38; p=0.046); GEE group×time p=0.035. | | NS: Child screen time (24-h media diary) - no significant between-group or within-group differences from baseline to 10 weeks (weekday/weekend). | NA |
| **DIET** | | | | | | | |
| Cunningham et al. (2026) [27] | NA | NS for child egg consumption or child dietary diversity | NA | NA | NA | NA | NA |
| Hunsrisakhun et al. (2025) [28] | NA | Within Group 1: S lower frequency of bottle feeds per day S lower frequency of times per week sleeping with bottle S lower frequency of times per week child | NS frequency of consuming sweet foods (times per day) Group 2 significantly increased frequency from baseline to 6 months (baseline mean=2.3 SD=1.6, 6 | NA | NA | NA | NA |

| | | | | | | | |
|---|---|---|---|---|---|---|---|
| | | waking at night to feed<br>S lower frequency child consuming milk or food after brushing teeth at night<br>NS adding sugar to food or drink for child<br>NS frequency consuming sweet food (times per day)<br><br>Within Group 2:<br>S lower frequency of bottle feeds per day<br>S lower frequency of times per week child waking at night to feed<br>S lower frequency child consuming milk or food after brushing teeth at night<br>S higher frequency consuming sweet foods (times per day)<br>NS all others<br><br>Between groups:<br>S lower frequency sleeping with bottle<br>NS all others | months mean = 2.6 SD=1.7) | | | | |
| **SLEEP** | | | | | | | |
| Mindell et al. (2011) [29] | NA | NA | NA | NA | NA | NA | S improvements compared to the control group |

PA, physical activity; SB, sedentary behavior; ST, screen time; NA, not assessed; NS not significant; S, significant; S/NS, some significant and some non-significant results.
[1] 12-35 months of age (≥1 to < 3 years).
[2] The combined interventions focused on multiple behaviors e.g., diet, and physical activity/sedentary behavior.
[3] This study focused on both the toddler period and preschool age.

**Table S7.** Details of the effectiveness of studies focusing on the preschool age[1] (n=8).

| Author (date), reference | Breastfeeding | Feeding practices | Diet | PA | SB | ST | Sleep |
|---|---|---|---|---|---|---|---|
| **COMBINED** | | | | | | | |
| Knowlden et al. (2015) [30] | NA | NA | S changes in fruit and vegetable consumption from pretest to post-test and from pretest to postintervention follow-up, suggesting an increase of 1.6 cups (95%CI: 0.7-2.5]) in the experimental group, relative to the control group (post hoc analyses). Significant changes in sugar-free beverage intake occurred from pretest to post-test and from pretest to postintervention follow-up, suggesting an overall increase of 1.1 cups of sugar-free beverages (95%CI: 0.3-1.8) in both groups over the duration of the intervention. | NA | NS | S changes in screen time from pretest to post-test and from pretest to postintervention follow-up, suggesting an overall decrease of 39 minutes (95%CI: -65.2 −12) of child screen time behavior in both groups over the duration of the intervention indicated by post-hoc analyses. | NA |
| Delisle-Nyström et al. (2017) [31] | NA | NA | S difference in the intake of sweetened beverages (-12 [SD 85] mL/d; 95%CI: 8-83) vs control | NS for MVPA | NS for sedentary time | NA | NA |
| Sun et al. (2017) [32] | NA | Small effect sizes were found for child-feeding practice related to restriction (d=0.27) | NS for child eating style, child eating related to hunger | NS for child activity level | NA | NA | NA |

| | | | | | | | |
|---|---|---|---|---|---|---|---|
| Hammersley et al. (2019) [33] | NA | S improvement in child feeding pressure to eat practices (-0.30, 95% CI 0.06 to -0.00) vs the control group | S reduced frequency of discretionary food intake (estimate -1.36,95% CI -2.27 to -0.45) vs the control group | NS for percentage activity (including sedentary time, LPA, MPA, VPA, and MVPA | NA | NS | NS |
| [37]**DIET** | | | | | | | |
| Bakirci-Taylor et al. (2018) [34] | NA | NA | S week x treatment interaction values for skin carotenoids (Veggie Meter values) and a S higher vegetable consumption (average difference 0.69 vegetables, +45 vs -0.29) in the intervention vs control group.<br><br>NS for frequency of fruits or vegetables | NA | NA | NA | NA |
| Hojati et al. (2024) [35] | NA | S between group factor on maternal nutrition knowledge (p<0.001, F=32.032, Ƞp2=0.230), feeding attitudes (p=0.037, F=4.450, Ƞp2=0.040) and nutrition practices (p=0.005, F=8.138, Ƞp2=0.071). | NA | NA | NA | NA | NA |
| **PHYSICAL ACTIVITY** | | | | | | | |
| Staiano et al. (2022) [36] | NA | NA | NA | NS for physical activity levels (LPA, MVPA, LPA+MVPA) | NS for SB | NA | NA |
| Phillips et al. (2026) [37] | NA | NA | NA | NS overall: did not significantly change proximal child or parent LPA/MVPA (60 min post-prompt). | NS overall: did not significantly change proximal sedentary time (60 min post-prompt) for children or parents. | NA | NA |

| | | | |
|---|---|---|---|
| | S (parents): MVPA increased over time across the 2-week period (intervention×day).<br><br>S (children, context-specific): MVPA increased when not with other adults and when weather pleasant vs too cold. | S (children, context-specific): sedentary time reduced when indoors at the time of the prompt. | |

PA, physical activity; SB, sedentary behavior; ST, screen time; NA, not assessed; NS not significant; S, significant; S/NS, some significant and some non-significant results; LPA, light physical activity; MPA, moderate physical activity; MVPA, moderate-to-vigorous physical activity; VPA, vigorous physical activity.

[1] 36-59 months of age (≥3 to < 5 years).

[2] The combined interventions focused on multiple behaviors e.g., diet, and physical activity/sedentary behavior.

**Table S8.** Co-design, process evaluation and engagement outcomes for interventions focusing on pregnancy to infancy (0-11 months) (n=24).

| Author (date), reference | Co-design/end user engagement | Intervention underpinned by theory | Process evaluation description | Engagement outcomes measured: Y/N (objective or subjective method) | Engagement outcomes: results | Analysis of impact of engagement on effectiveness: Y/N | Assessment of impact of engagement on the effectiveness: results |
|---|---|---|---|---|---|---|---|
| **BREASTFEEDING** | | | | | | | |
| Ahmed et al. (2016) [1] | Previous feasibility and acceptability study [39] collected mothers' views on the system usability and their perceptions of the system/intervention, but end-users were not involved in the development of the intervention messaging. | Self-regulation model from Bandura's Social Cognitive Theory | Prior feasibility and acceptability study [39] found the data entry for the monitoring system was not overly burdensome, and the researchers took steps to further improve feasibility by providing a mobile version, encouraging help from family, and offering technical support and reminders. | No | - | No | - |
| Unger et al. (2018) [2] | End-users (pregnant women and healthcare providers) were engaged through focus group discussions to design the content of the SMS messages. The intervention also incorporated features to address the end-users' | No | No formal process evaluation, but information about the implementation and engagement with the SMS platform suggest the intervention was feasible and acceptable to participants, with high levels of engagement and use of the two-way | Yes (objective) | Engagement outcomes were measured by tracking the number of messages delivered and received through the Mobile WACh SMS platform. The platform delivered over 3,000 messages to participants in the | No | - |

| | | | | | | | |
|---|---|---|---|---|---|---|---|
| | needs, such as providing the SMS messages at no cost to the participants and allowing for two-way communication between the participants and study staff. | | SMS component to address concerns between clinic visits. | | intervention arms and received over 1,100 messages, with an overall engagement rate of 83%. The engagement was also measured by tracking the number of messages sent by participants in the two-way SMS group, who were able to communicate with the study nurse about their concerns and questions. | | |
| Wu et al. (2020) [3] | The "Ke Xue Wei Yang (Optimal Feeding)" module was pretested with pregnant women in Huzhu County before being implemented in the study. | No | The 108 messages were published in the *Ke Xue Wei Yang* module were read >8892 times. | No | - | No | - |
| Lewkowitz et al. (2020) [4] | Initial app prototype was developed based on prior survey results, and then presented to multiple focus groups of low-income, predominantly black pregnant and postpartum women to incorporate their feedback. The final | No | 1) Feasibility, as measured by app usage and the proportion of participants who did not use their assigned app<br><br>2) Acceptability, as measured by participants' rating of the BFF app as the best breastfeeding | Yes (objective) | App app usage tracking, where a message was sent to a secure website each time the app was opened. The BFF app was opened a median of 15 times, while the control app was opened a median of 9 times, which was not a statistically significant | Yes | The paper found that engagement with the app did not impact its effectiveness in improving breastfeeding rates. Excluding women who did not use the app did not change the primary or secondary outcomes, and the difference in app |

| | | | | | | | |
|---|---|---|---|---|---|---|---|
| | product was unanimously approved by additional focus groups of the target population. | | resource at 6 weeks postpartum<br><br>The researchers used app usage data and participant self-report questionnaires to assess these process evaluation outcomes. | | difference. Additionally, more women in the BFF app group did not use their app compared to the control group, but this difference also did not reach statistical significance. | | usage between the intervention and control groups was not statistically significant. |
| Scott et al. (2021) [5] | The Milk Man app was based on a face-to-face intervention, and the decision to develop the Milk Man app was made based on the needs and preferences of the target audience (fathers). Both interventions were designed with input from the end users (fathers). | Social cognitive theory | Feasibility and acceptability of the Milk Man app was assessed through a comprehensive evaluation framework that examined the robustness of the technology, the intervention principles and engagement strategies, and the interaction of the user with the technology. The design and ease of use of the app were rated highly, and two-thirds of users indicated they would recommend the app to other fathers. | Yes (objective and subjective) | Engagement outcomes for the Milk Man app intervention were measured using a combination of app analytics data and self-reported data from the 6-week follow-up questionnaire. The app analytics data showed high levels of in-app interactions, with usage concentrated around the birth of the baby, and detailed metrics on engagement with the app's features like the library articles and conversation forum. The self-reported data from the questionnaire provided qualitative insights into how fathers used the app's features, | No | - |

| | | | | | | | |
|---|---|---|---|---|---|---|---|
| | | | | | particularly the online forum, for social support and information sharing. | | |
| Saucedo Baza et al. (2022) [6] | None reported | Theory of breastfeeding self-efficacy (grounded in social cognitive theory) | Acceptability and feasibility of the smartphone app were assessed via a questionnaire given to the intervention group participants, which asked about the usefulness of the application, ease of navigation, features they would like to see added, and how much time they spent using the application. | Yes (subjective) | Self-reported hours they spent using the app (estimated number of hours ranged from 1 to 10 ($M$ = 3.2, $SD$ = 2.5). | No | - |
| Doan et al. (2022) [7] | Formative research, including in-depth interviews and self-administered interviews with 22 mothers and 49 mothers, respectively, was conducted to identify key themes and inform the app's features. | Behavioral Intervention Technology Model and behavior change theories targeting breastfeeding self-efficacy | None reported | No | - | No | - |
| LeFevre et al. (2022) [8] | The intervention was designed and piloted in 2012-13 and then redesigned and scaled up. End-users also participated in | No | None reported | Yes (objective) | Call data records from the Kilkari IVR system were linked to the survey data to measure listening patterns for each subscriber, with | Yes | Higher engagement, as indicated by listening to ≥50% of the Kilkari content, was not associated with a significant impact |

| | | | | | | | |
|---|---|---|---|---|---|---|---|
| | qualitative interviews and cognitive interviews to refine the survey tools used to evaluate the intervention, | | | | "exposure" defined as listening to ≥50% of the cumulative duration of the calls related to a particular health outcome. "High listenership" rates, defined as the proportion of subscribers who listened to at least 50% or 75% of the total content of individual Kilkari calls, were stable over time with an average of 65% listening to more than 50% of the calls. | | on the primary outcome of exclusive breastfeeding. |
| Acar and Sahin (2023) [9] | End-users were involved in the development of the mobile application-based breastfeeding program through a pilot study with six primiparous mothers. The mothers provided feedback on technical issues with the application, which were then resolved by the mobile application developer. The researchers also engaged with the | No | Acceptability and satisfaction of the mobile application-based breastfeeding program (MABBP) from the perspective of the mothers who used it were assessed via a questionnaire (with both quantitative and qualitative questions). Mothers found the application design and usability to be acceptable, and they were satisfied with the | Yes (subjective) | 50% of mothers reported using the app ‘frequently’ | No | - |

| | | | | | | | |
|---|---|---|---|---|---|---|---|
| | mothers by answering their questions about the use of the application and providing them with instructions on how to use it. | | application's benefits, such as accessing reliable information, finding solutions to breastfeeding issues, and keeping a breastfeeding diary. | | | | |
| Hmone et al. (2023) [10] | End-users, including pregnant women, their family members, and other stakeholders, were engaged to inform the development of the text message intervention. The formative qualitative study directly shaped the research question, study design, and content of the text messages to address the perceived barriers and needs of the target population [40]. | Health Belief Model | None reported | No | - | No | - |
| Vila-Candel et al. (2024) [11] | None reported | No | None reported | Yes (objective) | Engagement outcomes were measured through the usage data provided by the LactApp application administrators, which included the number of consultations or entries made by | Yes | Higher engagement with the LactApp mobile app did not improve the effectiveness of the intervention in increasing breastfeeding rates at 6 months postpartum. The breastfeeding abandonment rate |

| | | | | | | | | |
|---|---|---|---|---|---|---|---|---|
| | | | | | participants in the application. Participants who made fewer than five entries in the application over 6 months were excluded, as this was considered the minimum level of adherence based on the average usage provided by the LactApp company. Additionally, non-adherence was more common among certain demographic groups. | | was similar between the control group and the intervention group, even when excluding the non-adherent participants from the intervention group. |
| Henshaw et al. (2024) [12] | None reported | Informed by a breastfeeding self-efficacy framework | None reported | No | - | No | - |
| De Mello et al. (2025) [13] | None reported | Breastfeeding self-efficacy | Follow up survey item: feedback regarding the mobile application. Results not specifically reported, however some suggestions for improvement from participants were mentioned briefly in the discussion. | Yes (subjective) | Follow up survey item asked whether or not the mobile application was utilized, results not reported. | No | - |
| Brown et al. (2025) [14] | The mHealth program was developed collaboratively with a multidisciplinary team including | Behavior change wheel and Theoretical Domains Framework | Acceptability was measured at 5 months via 3 items on 5-point Likert scale:<br>1. I found the program acceptable | Yes (objective and subjective) | Engagement click rates 16.3% overall across both doses.<br><br>Engagement opt-out rates = 6.8% overall. | No | - |

| | | | | | | | |
|---|---|---|---|---|---|---|---|
| | CFHS, multicultural health, Aboriginal partners, allied health professionals, and end-users (parents). | | 2. Since I started the program, I have been happy with how often I have received text messages<br>3. I would recommend the program to other caregivers<br><br>Acceptability strongly agree/agree responses (37% response rate)<br>1. High dose = 84% (181/215)<br>Low dose = 85% (218/255)<br>2. High dose = 85.5% (183/214)<br>Low dose = 90.5% (228/252)<br>3. High dose = 87%<br>Low dose = 86% (188/215) | | Sig higher opt-out rates in high-dose group (8.3%) compared to low-dose (5.3%).<br>Sig higher opt-out in high-dose group in phase 1 (7.0%), compared to low-dose (3.8%, p<0.001) | | |
| Cherie et al. (2025) [15] | Experts in behavior change and neonatal care reviewed and refined the text messages. | No | None reported | No | - | No | - |
| Gilano et al. (2025) [16] | None reported | Behavioral change communication frameworks | None reported | No | - | No | - |
| **BREASTFEEDING AND FEEDING PRACTICES** | | | | | | | |
| Palacios et al. (2018) [17] | Pediatricians and WIC personnel who work directly with the target low- | TransTheoretical Model (TTM) of health behavior change | 1) Feasibility of delivering the SMS messages, as indicated by the | Yes (objective) | 1) Whether the SMS messages were successfully delivered, as | No | - |

| | | | | | | | |
|---|---|---|---|---|---|---|---|
| | income, minority populations were consulted to ensure the cultural relevance and appropriateness of the intervention messages. Pediatricians and WIC registered dietitians also reviewed and provided feedback on the messages to align them with existing WIC messaging and practices [41]. | | successful delivery of all 18 messages, but with some participants experiencing issues with message delivery or opting out.<br><br>2) Acceptability and engagement of participants with the SMS intervention, as indicated by the decreasing response rates to questions sent via SMS over time. | | indicated by the number of participants who had messages bounce or opted out of receiving messages.<br><br>2) The response rate to SMS questions sent to participants every 2 weeks, which decreased over time from 52-55% for the first 4 questions to 32-34% for the last 3 questions. | | |
| Davis et al. (2023) [18] | Qualitative interviews with parents were conducted to understand their attitudes and preferences; this information was used to select the theoretical framework, and the text messages were then developed and piloted with parents before finalizing the intervention. | Health Belief Model | No formal process evaluation, but some qualitative insights into the acceptability and feasibility of the text messaging intervention are provided. The main issues identified were challenges with maintaining consistent contact with participants due to frequent changes in cell phone service, and the inability to blind participants to their group assignment, which could have influenced the outcomes. | No | - | No | - |

| | | | | | | | |
|---|---|---|---|---|---|---|---|
| Li et al. (2024) [19] | Key topics were generated through literature review and expert consultation. | WHO comprehensive health literacy model | None reported | Yes (objective) | Of 746 participants in the intervention group, 69.4% (518/746) had watched at least 1 video. | Yes | Within the intervention group, both participants who had not watched videos and those who watched at least 1 video demonstrated an increased total score. Using the generalized linear mixed model, compared to participants who watched at least 1 video, those in the control group had a significantly lower total score (β=2.68, 95% CI 0.20-5.17) and psychological score (β=1.72, 95% CI 0.13-3.32), after controlling for the baseline score and other potential confounders. |
| **COMBINED [2]** | | | | | | | |
| Wen et al. (2020) [20] | None reported | Health Belief Model | 1) The telephone intervention had limited reach, with only 61-87% of participants receiving the full set of 6 telephone sessions.<br><br>2) The SMS intervention had higher reach, with 96% of participants in the SMS group receiving the | Yes (objective) | Engagement outcomes were measured by tracking the number of participants who received each of the 6 scheduled telephone support sessions, as well as the number of participants who opted out of the SMS intervention. The telephone | No | - |

| | | | | | | | |
|---|---|---|---|---|---|---|---|
| | | | messages, though 15 participants opted out over the 12-month period. | | support sessions had varying levels of engagement, with only 61-87% of participants receiving the different sessions. The SMS intervention had a higher overall reach, with 96% of the SMS group receiving the messages, though 15 participants opted out by the end of the 12-month study. | | |
| Wu et al. (2023) [38] | The WeChat self-assessment questionnaire and feedback reports were pre-tested with 26 caregivers in the target communities, with their feedback and suggestions incorporated before finalizing the intervention. | No | Acceptability and perceived usefulness of the WeChat-based self-assessment tool, feedback report, and health education videos were assessed through a questionnaire administered to the caregivers at the second follow-up. | Yes (subjective) | Engagement outcomes were measured through a survey at the second follow-up, which found that the majority of participants (around 85%) filled out the self-assessment questionnaire at least 3 times, over 90% read the feedback report and found it useful, and nearly 80% watched the educational videos provided with the feedback report, with almost all of them finding the videos useful. | No | - |
| **DIET** | | | | | | | |

| | | | | | | | |
|---|---|---|---|---|---|---|---|
| Roed et al. (2021) [21] | Developed through a co-design process that involved end-users, including parents of toddlers and healthcare professionals, who provided input and feedback that was incorporated into the final intervention design and components. | Social cognitive theory | Feasibility and acceptability were assessed, specifically in terms of participant engagement with the website. The process evaluation found that 13% of invited participants did not enter the website at all. | Yes (objective) | Engagement outcomes were measured by tracking the number of lessons completed by participants in the intervention group on the Food4toddlers website. The number of participants out of 144 in the intervention group who completed the 22 lessons ranged from 21 (14.6%) to 87 (60.4%), with a general drop in lesson completion over time. 13% of invited participants did not enter the website at all. | No | - |
| Helle et al. (2019) [22] | None reported | Elements from attachment theory, social cognitive theory, and the framework of anticipatory guidance. | None reported | Yes (subjective) | At both the 12-month and 24-month time points, participants in the intervention group reported on how many of the infant feeding videos and cooking films they had watched. The majority (> 70%) reported watching most or all of the intervention content. | No | - |
| **SLEEP** | | | | | | | |
| Moon et al. (2017) [23] | End-users, including maternity | No | None reported | Yes (objective) | Engagement outcomes were | No | - |

| | | | | |
|---|---|---|---|---|
| | unit staff and family caregivers of newborn infants, were involved in the development of both the NQI and mHealth interventions. Qualitative feedback was collected from maternity unit staff and the target audience of family caregivers reviewed the educational content. | | measured by tracking whether participants viewed the initial videos and then received the subsequent daily and less frequent messages and videos over the 60-day intervention period, with the rates of participants opening and viewing the messages consistently higher than 50%. | |

**Table S9.** Co-design, process evaluation and engagement outcomes for interventions focusing on toddlers (12-35 months) (n=6).

| Author (date), reference | Co-design/end user engagement | Underpinned by theory | Process evaluation | Engagement outcomes measured (method) | Engagement outcomes: results | Analysis of impact of engagement on effectiveness | Assessment of impact of engagement on the effectiveness: results |
|---|---|---|---|---|---|---|---|
| **COMBINED [2]** | | | | | | | |
| Alexandrou et al. (2023) [3] [24] | Qualitative interviews with both parents (end-users) and child health care nurses (future implementers) were conducted to gather feedback and input on the app content and features. This feedback was used to adapt and translate the app into Somali, Arabic, and English to make it more accessible and culturally relevant for diverse families in Sweden. | Social cognitive theory and key behavior change techniques, such as shaping knowledge, goal setting, identification of barriers, self-monitoring of behavior, and feedback. | App usage and satisfaction via a self-report questionnaire: 79% of parents agreed they were satisfied with the app and the majority agreed that it was useful and utilized frequently. | Yes (subjective and objective) | Engagement outcomes measured through self-reported survey data from parents in the intervention group and objective app analytics data tracking how often parents used the app's registration feature to log their child's health behaviors: 54% of parents reported using the app ≥once a week and 67% reported taking part in the majority of the Themes; parents entered their child's health behaviors on average once/week during the 6-month intervention period for all 4 categories | No | - |
| Sandborg et al. (2025) [25] | None reported | Behavior change wheel | Not reported | Yes (objective) | 96% of participants logged in and used at least one app feature (n=523/547). Avg time spent in the app= 34 min | Yes | Spending more time in the app was associated with higher scores for total knowledge of movement behaviors (β =0.56; |

| | |
|---|---|
| (SD 37; range 0–270 min)<br>Avg number of completed modules= 2.8 (SD 3.1; range 0–8) | 95%CI 0.23 to 0.89; P= 0.001) and physical activity knowledge (β =0.21; 95%CI: 0.07 to 0.36; P= 0.005), ease of parenting (β =0.13; 95%CI: 0.02 to 0.24; P= 0.028), as well as total score for family rules (β =0.51; 95%CI: 0.04 to 0.98; P= 0.035) and rules for screen time (β =0.22; 95%CI: 0.02 to 0.43; P= 0.031). Completing more modules was associated with higher scores for parental knowledge of movement behaviors (β =0.09; 95%CI: 0.02 to 0.15; P= 0.008) and physical activity (β =0.03; 95%CI: 0.00 to 0.06; P= 0.027), confidence (β =0.18; 95%CI: 0.02 to 0.35; P= 0.026), and total score for family rules (β =0.11; 95%CI: 0.02 to 0.20; P= 0.019), as well as less parental screen time (β |

| | | | | | | | |
|---|---|---|---|---|---|---|---|
| | | | | | | | =−0.08 h/day; 95%CI: −0.16 to −0.00; P= 0.04, equivalent to 5 min less per day). |
| Jongpaiboonpatana et al. (2025) [26] | None reported | Social learning theory | Not reported | No | - | No | - |
| **DIET** | | | | | | | |
| Cunningham et al. (2026) [27] | Before implementation of a similar text message campaign, messages were pre-tested among 1000-day mothers in two neighboring districts. | A detailed theory of change was developed as part of the intervention design. | Not reported | Yes (subjective) | 31% (n=381) of mothers in intervention group received and opened messages. | Yes | The intervention had a positive effect in intervention households where text messages were received and opened, compared to those who did not receive, or did not open messages: S higher child egg consumption (AOR= 1.41, 95% CI: 1.03-1.93, p=0.030) S higher minimum dietary diversity (AOR=1.36, 95% CI: 1.07-1.73, p=0.011) |
| Hunsrisakhun et al. (2025) [28] | Both chatbots were developed and pilot-tested in 30 participants. | Protection motivation theory, and artificial Intelligence chatbot behavior change model. | Satisfaction survey administered at 3-month follow-up (0=very dissatisfied, 5=very satisfied). Mean satisfaction level 4.0 in both groups, no sig differences. | Yes (objective) | Participants in Group I had an average engagement with the chatbot of 13.8 ± 8.2 days out of 21 days, and participants in Group II had an average usage rate of the chatbot of 15.4 ± 7.5 days out of 21 days. | No | - |
| **SLEEP** | | | | | | | |

| | | | | | | | |
|---|---|---|---|---|---|---|---|
| Mindell et al. (2011) [29] | None reported | No | Acceptability and satisfaction via a self-report questionnaire, which found that the majority of mothers found the individualized recommendations to be helpful, were likely to continue using them, and felt more confident in managing their child's sleep. | No | - | No | - |

**Table S10.** Co-design, process evaluation and engagement outcomes for interventions focusing on preschoolers (36–59 months) (n=8).

| **Author (date), reference** | **Co-design/end user engagement** | **Underpinned by theory** | **Process evaluation** | **Engagement outcomes measured (method)** | **Engagement outcomes: results** | **Analysis of impact of engagement on effectiveness** | **Assessment of impact of engagement on the effectiveness: results** |
|---|---|---|---|---|---|---|---|
| **COMBINED [2]** | | | | | | | |
| Knowlden et al. (2015) [30] | None reported | Social Cognitive Theory | Measures of program fidelity, dose delivered, dose received, reach, recruitment, and context were assessed through telephone counselling and online surveys. Results indicated that both the EMPOWER and Healthy Lifestyles interventions exceeded the 0.90 level of implementation excellence for these measures, and there were no significant differences between the two groups in terms of dose satisfaction and recruitment time. | No | - | No | - |
| Delisle-Nyström et al. (2017) [31] | Content was first tested in a pilot study with 10 parents of a 4-year-old child then, after reiteration of the intervention based on parents' | Social cognitive theory | 1) Mobile app was highly accessible and usable for the parents, with nearly all of them able to download and use the app without technical | Yes (objective) | The engagement outcomes for the MINISTOP intervention were measured by tracking the number of feedback messages | No | - |

| | | | | | | | |
|---|---|---|---|---|---|---|---|
| | comments (obtained via a questionnaire), an additional 19 parents with a 4-year-old child were recruited and user tested a demonstration version of the MINISTOP app, providing comments on both content and feasibility [42]. | | issues.<br><br>2) Parents demonstrated high levels of engagement and sustained usage of the mobile application, with many recording the requested parameters well beyond the minimum requirement. | | read by parents and the number of recordings they made for various dietary and physical activity behaviors within the MINISTOP app. Approximately 60% of parents used the iOS version of the application and no technical problems were reported for either iOS or Android users. Additionally, 25% of families recorded the 4 parameters (fruits, candy, sweetened beverages, and sedentary behavior) for at least 130 days out of the maximum 168 days. | | |
| Sun et al. (2017) [32] | End-users (Chinese mothers with preschool-aged children) were involved in the development of the intervention through a focus group, providing feedback on the format, content, usability, and cultural appropriateness of | Information Motivation Behavioral Skills (IMB) model | 1) Post-intervention satisfaction interviews with a sample of participants to assess the acceptability and satisfaction with the intervention<br><br>2) Identification of factors that contributed to the | No | - | No | - |

| | | | | | | | |
|---|---|---|---|---|---|---|---|
| | the intervention materials, with changes made based on this feedback. A song with the key messages was incorporated based on feedback from the focus group to help with information retention and motivation. | | success of the intervention, including the feasibility and acceptability of the tablet-based format, the cultural and linguistic tailoring, and the use of engaging multimedia content | | | | |
| Hammersley et al. (2019) [33] | Intervention was based on formative research [43] with parents of preschool-aged children (n=27) and piloted in 47 parent-child dyads [44] | Social cognitive theory | Acceptability and user satisfaction was assessed through a questionnaire completed by 38 out of the 42 participants (90%) in the intervention group, which evaluated participants' perceptions of the program content, length, goal-setting, Facebook group, and overall modality. Most participants agreed or strongly agreed that the program content was interesting, easy to understand, and relevant, and that the length and goal-setting were appropriate. However, only | Yes (objective and subjective) | 1) Tracking the number of intervention program modules completed by participants, with at least 5 of 6 modules completed by 69% of participants.<br><br>2) Self-reported discussion of the program with extended family members, with 74% of participants reporting they discussed the program with family.<br><br>3) Self-reported feedback on the usefulness of the Facebook group component, with only 39% of participants | No | - |

| | | | | | | | |
|---|---|---|---|---|---|---|---|
| | | | 39% of participants agreed that the Facebook component was useful. | | agreeing or strongly agreeing that it was useful. | | |
| **DIET** | | | | | | | |
| Bakirci-Taylor et al. (2019) [34] | None reported | Social Cognitive Theory | 1) Acceptability of text messages (both intervention and control groups)<br><br>2) Reactions to the mobile Jump2Health website (intervention group only)<br><br>3) Reactions to the Facebook page (intervention group only)<br><br>Survey results indicated that participants engaged more with the Facebook page than the mobile website, and provided insights into what content and features of the Facebook page were most appealing. The survey also revealed that some participants found the mobile website to be less user-friendly and engaging compared | Yes (objective) | 1) Facebook page analytics, including reach (number of times posts entered users' screens) and engagement (likes, comments, and click-through rates)<br><br>2) The number of participants who created an account on the password-protected mobile Jump2Health website | No | - |

| | | | | | | | |
|---|---|---|---|---|---|---|---|
| | | | to the Facebook page. | | | | |
| Hojati et al. (2024) [35] | The MyKid'sNutrition app was designed and developed by a team of nutrition experts, pediatric specialists, designers and developers. | No | No | - | No | No | - |
| **PHYSICAL ACTIVITY** | | | | | | | |
| Staiano et al. (2022) [36] | None reported | Social cognitive theory | Feasibility (adherence) was measured by the number of activity periods self-reported as complete by participants. Acceptability was measured through parent-reported surveys at weeks 4, 8, and 12 on satisfaction, helpfulness, ease of use, and likelihood to recommend the app, as well as the System Usability Scale at week 12. | Yes (subjective) | Parents self-reported the number of 12-minute activity periods complete by selecting stars in the app. On average, parents reported completing 47 out of 60 prescribed activity breaks (564 minutes total), with similar adherence rates between the Motor Skills (71%) and Free Play (87%) groups. | No | - |
| Phillips et al. (2026) [37] | None reported | Self-determination theory | Acceptability was measured post-intervention with 3 purpose-designed questions (1=completely disagree, 5=completely agree):<br>1. Intervention taught me | No | - | No | - |

| | | | |
|---|---|---|---|
| | | something new about getting my child active (Mean 3.59 SD .83)<br>2. Intervention helped get my child to be more active (Mean 3.45, SD .94)<br>3. Intervention helped get me to be more active (Mean 3.36 SD 1.02) | |

## References

1. Ahmed AH, Roumani AM, Szucs K, Zhang L, King D. The Effect of Interactive Web-Based Monitoring on Breastfeeding Exclusivity, Intensity, and Duration in Healthy, Term Infants After Hospital Discharge. *JOGNN - Journal of Obstetric, Gynecologic, and Neonatal Nursing*. 2016;45(2):143-154. doi:10.1016/j.jogn.2015.12.001

2. Unger JA, Ronen K, Perrier T, et al. Short message service communication improves exclusive breastfeeding and early postpartum contraception in a low- to middle-income country setting: a randomised trial. *BJOG*. 2018;125(12):1620-1629. doi:10.1111/1471-0528.15337

3. Wu Q, Huang Y, Liao Z, van Velthoven MH, Wang W, Zhang Y. Effectiveness of wechat for improving exclusive breastfeeding in Huzhu county China: Randomized controlled trial. *J Med Internet Res*. 2020;22(12):e23273. doi:10.2196/23273

4. Lewkowitz AK, López JD, Carter EB, et al. Impact of a novel smartphone application on low-income, first-time mothers' breastfeeding rates: a randomized controlled trial. *Am J Obstet Gynecol MFM*. 2020;2(3). doi:10.1016/J.AJOGMF.2020.100143

5. Scott JA, Burns SK, Hauck YL, et al. Impact of a Face-To-Face Versus Smartphone App Versus Combined Breastfeeding Intervention Targeting Fathers: Randomized Controlled Trial. *JMIR Pediatr Parent*. 2021;4(2):e24579. doi:10.2196/24579

6. Saucedo Baza A, Mignacca C, Delgado PE, et al. A Technological Approach to Improved Breastfeeding Rates and Self-Efficacy: A Randomized Controlled Pilot Study. *https://doi.org/101177/08903344231190625*. 2023;39(4):679-687. doi:10.1177/08903344231190625

7. Doan TTD, Pham NM, Binns C, et al. Effect of a Mobile Application on Breastfeeding Rates Among Mothers Who Have Cesarean Deliveries: A Randomized Controlled Trial. *Breastfeed Med*. 2022;17(10):832-840. doi:10.1089/BFM.2022.0088

8. Lefevre AE, Shah N, Scott K, et al. The impact of a direct to beneficiary mobile communication program on reproductive and child health outcomes: a randomised controlled trial in India. *BMJ Glob Health*. 2022;6(Suppl 5). doi:10.1136/BMJGH-2022-008838

9. Acar Z, Şahin N. Development of a mobile application -based breastfeeding program and evaluation of its effectiveness. *J Pediatr Nurs*. 2024;74:51-60. doi:10.1016/J.PEDN.2023.11.011

10. Hmone MP, Li M, Agho KE, Alam NA, Chad N, Dibley MJ. Tailored text messages to improve breastfeeding practices in Yangon, Myanmar: the M528 individually randomized controlled trial. *Am J Clin Nutr*. 2023;117(3):518-528. doi:10.1016/J.AJCNUT.2023.01.003

11. Vila-Candel R, Mena-Tudela D, Franco-Antonio C, Quesada JA, Soriano-Vidal FJ. Effects of a mobile application on breastfeeding maintenance in the first 6 months after

birth: Randomised controlled trial (COMLACT study). *Midwifery*. 2024;128:103874. doi:10.1016/J.MIDW.2023.103874

12. Henshaw E, Cooper M, Wood T, Krishna S, Lockhart M, Doan S. A randomized controlled trial of the Happy, Healthy, Loved personalized text-message program for new parent couples: impact on breastfeeding self-efficacy and mood. *BMC Pregnancy and Childbirth 2024 24:1*. 2024;24(1):506-. doi:10.1186/s12884-024-06684-9
13. de Mello Sa SR, Wang Z, Sapkalova V, et al. A smartphone-based application to improve breastfeeding duration and self-efficacy: a randomized controlled clinical trial. *Women Health*. 2025;65(2):154-166. doi:10.1080/03630242.2024.2448519
14. Brown AL, Hudson N, Pinfold J, et al. The Impact of Dose in an mHealth Intervention to Support Parents and Carers Via Healthy Beginnings for Hunter New England Kids Program: Pragmatic Randomized Controlled Trial. *JMIR Form Res*. 2025;9(1):e70158. doi:10.2196/70158
15. Cherie N, Wordofa MA, Debelew GT. The effect of an interactive mobile health intervention to improve community-based essential neonatal care practices among postpartum women in northeast Ethiopia: a cluster randomized controlled trial. *Int Health*. 2025;17(5):820-835. doi:10.1093/inthealth/ihae080
16. Gilano G, Dekker A, Fijten R. The Effect of mHealth on Exclusive Breastfeeding and Its Associated Factors Among Women in South Ethiopia: A Cluster Randomized Controlled Trial. *Nutrients 2025, Vol 17, Page 3477*. 2025;17(21):3477. doi:10.3390/nu17213477
17. Palacios C, Campos M, Gibby C, Meléndez M, Lee JE, Banna J. Effect of a Multi-Site Trial using Short Message Service (SMS) on Infant Feeding Practices and Weight Gain in Low-Income Minorities. *J Am Coll Nutr*. 2018;37(7):605-613. doi:10.1080/07315724.2018.1454353
18. Davis KE, Klingenberg A, Massey-Stokes M, et al. The Baby Bites Text Messaging Project with randomized controlled trial: texting to improve infant feeding practices. *Mhealth*. 2023;9:11. doi:10.21037/MHEALTH-22-31/COIF
19. Li Y, Xiao Q, Chen M, et al. Improving Parental Health Literacy in Primary Caregivers of 0- to 3-Year-Old Children Through a WeChat Official Account: Cluster Randomized Controlled Trial. *JMIR Public Health Surveill*. 2024;10(1):e54623. doi:10.2196/54623
20. Wen LM, Rissel C, Xu H, et al. Effects of Telephone and Short Message Service Support on Infant Feeding Practices, "Tummy Time," and Screen Time at 6 and 12 Months of Child Age: A 3-Group Randomized Clinical Trial. *JAMA Pediatr*. 2020;174(7):657-664. doi:10.1001/JAMAPEDIATRICS.2020.0215

21. Røed M, Medin AC, Vik FN, et al. Effect of a parent-focused eHealth intervention on children's fruit, vegetable, and discretionary food intake (Food4toddlers): Randomized controlled trial. *J Med Internet Res*. 2021;23(2):e18311. doi:10.2196/18311

22. Helle C, Hillesund ER, Wills AK, Øverby NC. Evaluation of an eHealth intervention aiming to promote healthy food habits from infancy -the Norwegian randomized controlled trial Early Food for Future Health. *Int J Behav Nutr Phys Act*. 2019;16(1). doi:10.1186/S12966-018-0763-4

23. Moon RY, Hauck FR, Colson ER, et al. The Effect of Nursing Quality Improvement and Mobile Health Interventions on Infant Sleep Practices: A Randomized Clinical Trial. *JAMA*. 2017;318(4):351-359. doi:10.1001/JAMA.2017.8982

24. Alexandrou C, Henriksson H, Henström M, et al. Effectiveness of a Smartphone App (MINISTOP 2.0) integrated in primary child health care to promote healthy diet and physical activity behaviors and prevent obesity in preschool-aged children: randomized controlled trial. *Int J Behav Nutr Phys Act*. 2023;20(1). doi:10.1186/S12966-023-01405-5

25. Sandborg J, Downing KL, Orellana L, et al. Six-month intervention effect of a digital movement behavior intervention on parent- and child intermediary outcomes—results from the Let's Grow randomized controlled trial. *International Journal of Behavioral Nutrition and Physical Activity* . 2025;22(1):1-14. doi:10.1186/S12966-025-01764-1/TABLES/3

26. Jongpaiboonpatana P, Jirakran K, Trairatvorakul P, Chonchaiya W. Increasing parent–child play frequency via exclusively online, video-based, peer-to-peer modeling program: randomized controlled trial. *Pediatric Research 2025 98:3*. 2025;98(3):997-1005. doi:10.1038/s41390-025-03800-z

27. Cunningham K, Cech S, Gupta A Sen, Rana PP, Humphries D, Frongillo EA. Text messages to improve young child diets: Results from a cluster-randomized controlled trial in Kanchanpur, Nepal. *Matern Child Nutr*. 2026;22(1):e13702. doi:10.1111/mcn.13702

28. Hunsrisakhun J, Naorungroj S, Tangkuptanon W, Wattanasit P, Pupong K, Pithpornchaiyakul S. Impact of Oral Health Chatbot With and Without Toothbrushing Training on Childhood Caries. *Int Dent J*. 2025;75(2):1348-1359. doi:10.1016/j.identj.2024.09.028

29. Mindell JA, Du Mond CE, Sadeh A, Telofski LS, Kulkarni N, Gunn E. Efficacy of an internet-based intervention for infant and toddler sleep disturbances. *Sleep*. 2011;34(4). doi:10.1093/SLEEP/34.4.451

30. Knowlden AP, Sharma M, Cottrell RR, Wilson BRA, Johnson ML. Impact evaluation of Enabling Mothers to Prevent Pediatric Obesity through Web-Based Education and Reciprocal Determinism (EMPOWER) Randomized Control Trial. *Health Educ Behav*. 2015;42(2):171-184. doi:10.1177/1090198114547816

31. Nyström CD, Sandin S, Henriksson P, et al. Mobile-based intervention intended to stop obesity in preschool-aged children: The MINISTOP randomized controlled trial. *American Journal of Clinical Nutrition*. 2017;105(6):1327-1335. doi:10.3945/ajcn.116.150995

32. Sun A, Cheng J, Bui Q, Liang Y, Ng T, Chen JL. Home-Based and Technology-Centered Childhood Obesity Prevention for Chinese Mothers With Preschool-Aged Children. *https://doi-org.proxy.kib.ki.se/101177/1043659617719139*. 2017;28(6):616-624. doi:10.1177/1043659617719139

33. Hammersley ML, Okely AD, Batterham MJ, Jones RA. An internet-based childhood obesity prevention program (TIMe2bhealthy) for parents of preschool-aged children: Randomized controlled trial. *J Med Internet Res*. 2019;21(2). doi:10.2196/11964

34. Bakırcı-Taylor AL, Reed DB, McCool B, Dawson JA. mHealth Improved Fruit and Vegetable Accessibility and Intake in Young Children. *J Nutr Educ Behav*. 2019;51(5):556-566. doi:10.1016/J.JNEB.2018.11.008

35. Hojati A, Farhangi MA. MyKid'sNutrition mobile application: effect on mothers' nutritional knowledge and nutritional status of preschool-aged children with undernutrition – a randomised controlled trial. *BMJ Nutr Prev Health*. 2025;8(1):29-37. doi:10.1136/bmjnph-2024-001007

36. Staiano AE, Newton RL, Beyl RA, et al. mHealth Intervention for Motor Skills: A Randomized Controlled Trial. *Pediatrics*. 2022;149(5). doi:10.1542/PEDS.2021-053362/186761

37. Phillips SM, Bourke M, Inniss B V., Ahluwalia M, Tucker P. A pilot effectiveness study of a just-in-time micro-randomized controlled trial on the physical activity and sedentary time of young children and their parents: The active family m-health intervention. *PLoS One*. 2026;21(1):e0340687. doi:10.1371/journal.pone.0340687

38. Wu Q, Wang X, Zhang J, Zhang Y, van Velthoven MH. The effectiveness of a WeChat-based self-assessment with a tailored feedback report on improving complementary feeding and movement behaviour of children aged 6–20 months in rural China: a cluster randomized controlled trial. *Lancet Reg Health West Pac*. 2023;37:100796. doi:10.1016/J.LANWPC.2023.100796

39. Ahmed AH, Ouzzani M. Interactive web-based breastfeeding monitoring: feasibility, usability, and acceptability. *J Hum Lact*. 2012;28(4):468-475. doi:10.1177/0890334412451869

40. Hmone MP, Dibley MJ, Li M, Alam A. A formative study to inform mHealth based randomized controlled trial intervention to promote exclusive breastfeeding practices in Myanmar: incorporating qualitative study findings. *BMC Med Inform Decis Mak*. 2016;16(1). doi:10.1186/S12911-016-0301-8

41. Banna J, Campos M, Gibby C, et al. Multi-site trial using short mobile messages (SMS) to improve infant weight in low-income minorities: Development, implementation, lessons learned and future applications. *Contemp Clin Trials*. 2017;62:56-60. doi:10.1016/J.CCT.2017.08.011

42. Delisle C, Sandin S, Forsum E, et al. A web- and mobile phone-based intervention to prevent obesity in 4-year-olds (MINISTOP): a population-based randomized controlled trial. *BMC Public Health*. 2015;15(1). doi:10.1186/S12889-015-1444-8

43. Jones RA, Price N, Okely AD, Lockyer L. Developing an online program to prevent obesity in preschool-aged children: What do parents recommend? *Nutrition & Dietetics*. 2009;66(3):151-157. doi:10.1111/J.1747-0080.2009.01359.X

44. Jones R, Wells M, Okely A, Lockyer L, Walton K. Is an online healthy lifestyles program acceptable for parents of preschool children? *Nutrition & Dietetics*. 2011;68(2):149-154. doi:10.1111/J.1747-0080.2011.01514.X
